# Presynaptic perspective: Axonal transport defects in neurodevelopmental disorders

**DOI:** 10.1083/jcb.202401145

**Published:** 2024-04-03

**Authors:** Gui-Jing Xiong, Zu-Hang Sheng

**Affiliations:** 1 Synaptic Function Section, The Porter Neuroscience Research Center, National Institute of Neurological Disorders and Stroke, National Institutes of Health, Bethesda, MD, USA

## Abstract

Disruption of synapse assembly and maturation leads to a broad spectrum of neurodevelopmental disorders. Presynaptic proteins are largely synthesized in the soma, where they are packaged into precursor vesicles and transported into distal axons to ensure precise assembly and maintenance of presynapses. Due to their morphological features, neurons face challenges in the delivery of presynaptic cargos to nascent boutons. Thus, targeted axonal transport is vital to build functional synapses. A growing number of mutations in genes encoding the transport machinery have been linked to neurodevelopmental disorders. Emerging lines of evidence have started to uncover presynaptic mechanisms underlying axonal transport defects, thus broadening the view of neurodevelopmental disorders beyond postsynaptic mechanisms. In this review, we discuss presynaptic perspectives of neurodevelopmental disorders by focusing on impaired axonal transport and disturbed assembly and maintenance of presynapses. We also discuss potential strategies for restoring axonal transport as an early therapeutic intervention.

## Teaser

Xiong and Sheng review recent advances in presynaptic mechanisms of neurodevelopmental disorders by focusing on impaired axonal transport of presynaptic cargos.

## Introduction

The human central nervous system (CNS) consists of hundreds of thousands of interconnected neuronal circuits that give rise to perception, cognition, and behavior. These circuits are wired together through the formation of ∼10^15^ synapses between ∼10^12^ neurons (Herculano-Houzel, 2012). Intense synaptogenesis occurs during embryonic and early postnatal stages, persisting throughout adolescence and even into the third decade of human life (Petanjek et al., 2011). Any disruption of synapse assembly, maturation, or remodeling leads to a broad spectrum of neurodevelopmental disorders (NDDs) characterized by an inability to reach cognitive, emotional, and motor developmental milestones, including autism spectrum disorders (ASDs), attention-deficit/hyperactivity disorder, intellectual disability (ID), disorders in communication, learning, and motor function, developmental epilepsies, and schizophrenia (SZ) (Sydnor et al., 2021; Thapar et al., 2017). NDD prevalence has progressively increased over the past decades; an estimated one in six children aged 3–17 years old in the United States have one form of NDD (Zablotsky et al., 2019). Despite the broad genetic heterogeneity of NDDs, genetic studies have revealed many human NDD-linked variants in genes associated with synapse formation and function, leading to a central hypothesis that synaptic pathology is one of the major causative mechanisms underlying the etiology of NDDs (Grant, 2012).

Synapse formation (or synaptogenesis) is a multistage process in the assembly of specialized synaptic structures, including (1) axon and dendrite contacts through dynamic filopodia, (2) synaptic cargo transport from the soma to nascent synapses, (3) recruitment and assembly of these synaptic components at newly forming synapses, and (4) maintenance and remodeling of synapses (Chia et al., 2013; Jin and Garner, 2008; Südhof, 2021). Deleterious variants in genes encoding cell-adhesion molecules (CAMs) and scaffolding proteins located at the postsynaptic density significantly alter the course of brain development, and therefore it is not surprising that they have been repeatedly associated with NDDs (Bourgeron, 2015; Exposito-Alonso and Rico, 2022; Michetti et al., 2022; Parenti et al., 2020). In contrast, little is known about the impact of axonal transport of presynaptic components on the etiology of NDDs, largely due to challenges in characterizing transient and dynamic transport events in live neurons. Recent studies have started to decipher genetic variants in the axonal transport machinery that may contribute to presynaptic mechanisms underlying NDDs (Rizalar et al., 2021).

**Figure 1.**
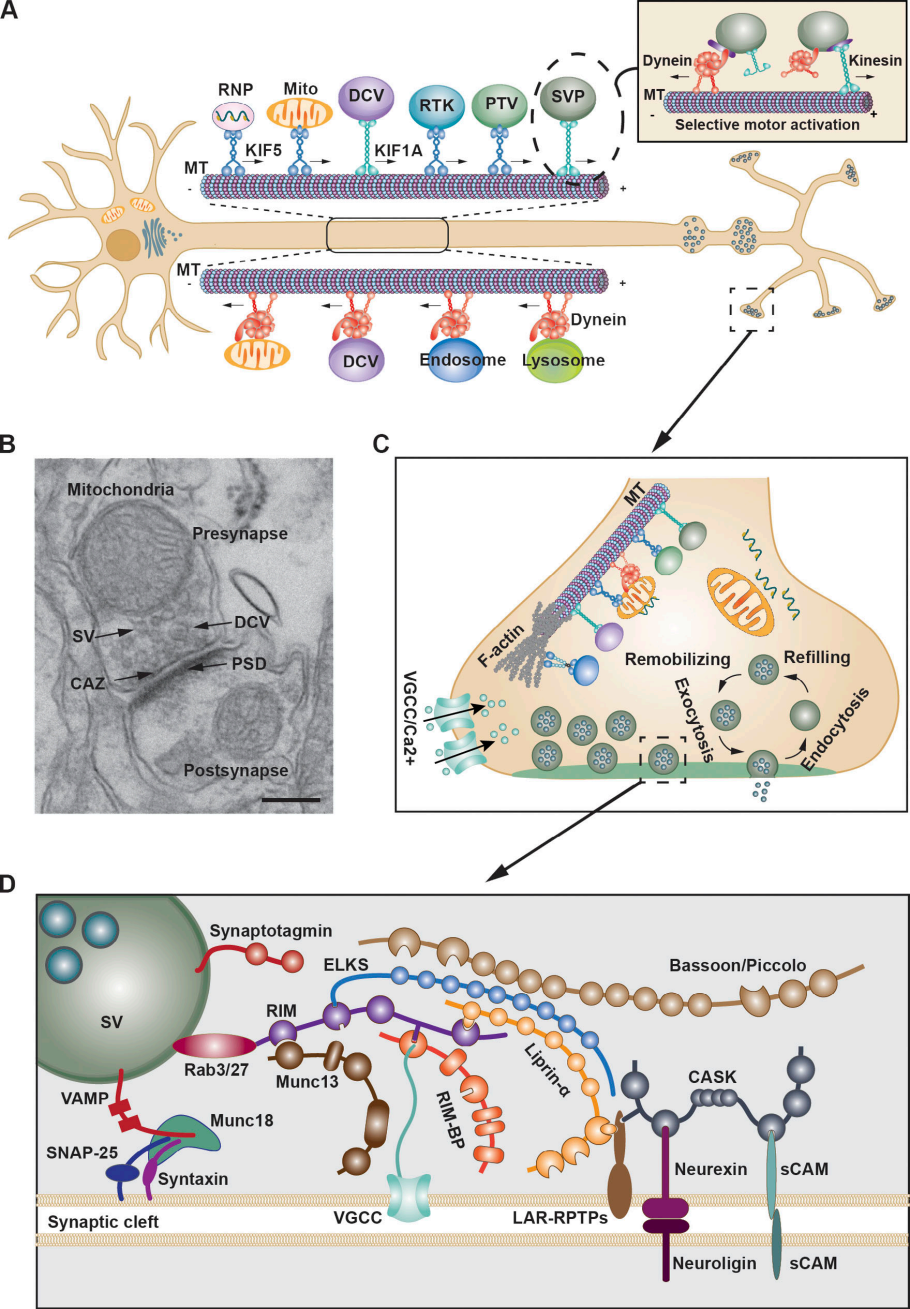
**Axonal transport in presynaptic assembly and maintenance. (A)** Schematic of the bidirectional axonal transport essential for presynapse assembly and maintenance. Major presynaptic proteins are synthesized in the cell body and transit the Golgi apparatus where they are sorted into presynaptic cargos, including SVPs, PTVs, DCVs, and RTK-carrying vesicles. These cargos are delivered along axonal MT tracks by kinesin motors to presynaptic terminals. SEs, mitochondria, and endo-lysosomes carrying defective proteins undergo dynein-driven retrograde transport toward the soma for retrograde signaling, degradation, or turnover. The insert indicates that both kinesin and dynein motors can be recruited to presynaptic cargos and their net favored transport direction is determined by the differentially activated motor state. **(B)** Electron micrograph showing pre- and postsynaptic specializations from mouse hippocampal slices in CA1 regions. Scale bar: 200 nm. **(C)** Transported cargos are released to build a presynapse where SVPs give rise to mature SVs and proteins are assembled into CAZ. Mitochondria are recruited to provide ATP and modulate Ca^2+^ signals. mRNAs serve as a platform to replenish presynaptic proteins locally. The presynaptic F-actin networks are required for SV docking and mobilization. **(D)** Schematic of presynaptic components, including sCAMs (neurexin, neuroligin, and LRPTP), CAZ proteins (ELKS, CASK, RIM, RIM-RBP, liprin-α, Munc13, Piccolo, and Bassoon), VGCCs, and SV fusion machinery (VAMP, SNAP25, Syntaxin, Synaptotagmin, Munc18, and Rab3). (Note that not all interactions are displayed. Sizes of molecules and cargoes are only for illustrative purposes and are not drawn to scale).

Presynaptic proteins are largely synthesized in the soma where they are packaged into precursor vesicles and then anterogradely transported into nascent synapses along axonal microtubules (MTs) by kinesin motors. Defective presynaptic organelles and proteins are retrogradely transported toward the soma by dynein motors for degradation or turnover (Fig. 1 A). Amazingly, human motor neuron axons can extend up to 1 m long with extensive terminal branching, and thus presynaptic terminals are positioned far away from the cell body. Due to these morphological features, neurons face exceptional challenges for the targeted delivery of presynaptic components along such long axons. Several adaptors and scaffolding proteins assist motor proteins in driving the bidirectional transport of synaptic cargos to ensure precise assembly, maintenance, and remodeling of presynapses (Cai et al., 2007; Guedes-Dias and Holzbaur, 2019). A growing number of mutations in genes encoding the transport machinery have been associated with NDDs (Hirokawa et al., 2010; Sleigh et al., 2019; Xiong et al., 2021). These findings have broadened our view beyond the scope of postsynaptic mechanisms. Thus, elucidating presynaptic mechanisms of NDDs is an important emerging frontier. In this review, we limit our discussion to presynaptic perspectives of NDDs by focusing on impaired axonal transport of presynaptic cargos. First, we provide an overview of axonal transport machineries and mechanisms driving targeted axonal transport of various presynaptic cargos to ensure the stoichiometric assembly and maintenance of presynaptic active zones (AZs). Second, we examine current literature emerging from human and mouse studies revealing genetic mutations in genes encoding transport machineries that associate with a wide range of NDDs. Finally, we discuss perspectives on the potential strategies for restoring axonal transport as an early therapeutic intervention for NDDs.

## Presynaptic assembly and maintenance

Synapses are highly asymmetric intercellular junctions composed of a presynaptic terminal and a juxtaposed postsynaptic density, separated by the synaptic cleft, where trans-synaptic CAMs provide connections between pre- and postsynaptic membranes (Fig. 1 B). Presynaptic terminals (or boutons) are established from the axonal growth cone and along axonal segments, which allow one axon to form enormous *en passant* synaptic connections with many dendrites along its route. A single rat hippocampal CA3 mossy/pyramidal neuron makes ∼40,000 synapses over ∼0.2 m length of axon (Buckmaster et al., 1996; Ishizuka et al., 1990; Li et al., 1994). The presynaptic compartment contains hundreds of neurotransmitter-filled synaptic vesicles (SVs) and a network of scaffolding proteins known as the cytomatrix of active zones (CAZs), a patterned structure with multiple protein complexes that (1) serves as the platform for recruiting and anchoring SVs at AZ release sites, (2) enables the physical coupling of voltage-gated calcium channels (VGCCs) to SV fusion sites, and (3) renders SV fusion competent by facilitating SNARE complex formation (Fig. 1, C and D). With this sophisticated protein machinery, SV exocytosis is executed within less than 1 ms upon VGCC opening in response to action potential firing (Südhof, 2012).

These presynaptic components are mainly synthesized in the soma and anterogradely transported along lengthy axonal MTs into distal presynaptic sites. While membranous presynaptic components (such as CAMs, VGCCs, and SV proteins) are synthesized by the rough endoplasmic reticulum in the soma and exported to the trans-Golgi network, non-membrane CAZ proteins are mainly synthesized on cytoplasmic ribosomes in the soma before undergoing axonal transport (Maas et al., 2012; Shapira et al., 2003; Zhai et al., 2001). Upon arrival at nascent synapses, these transported cargos are unloaded from motors and captured for assembly of AZ structures through multiple protein interactions (Fig. 1 D). AZ-like condensates may form by phase separation through multiple low-affinity interactions (Wu et al., 2019), although these condensates have not been validated at synapses *in vivo*.

Once a synapse is established, its structure and function are highly plastic with neuronal development and undergo activity-dependent remodeling. Existing synaptic connections can be strengthened, weakened, or eliminated, allowing the brain to adjust and optimize synaptic responses throughout life (Südhof, 2021). In response to appropriate signals, large (75–100 nm) dense-core vesicles (DCVs) and signaling endosomes (SEs) containing neuropeptides and neurotrophins, respectively, are transported anterogradely or retrogradely along the axon to regulate synapse maturation and synaptic strength (Harrington and Ginty, 2013; Wong et al., 2012). Studies combining *in vivo* metabolic labeling and mass spectrometry suggest that turnover rates of presynaptic proteins can range from 5 h or less to more than 50 days (Cohen et al., 2013; Fornasiero et al., 2018; Truckenbrodt et al., 2018). Thus, the maintenance of presynapses requires continual anterograde transport and replenishment of new presynaptic components and retrograde transport of defective or aged presynaptic components toward the soma for turnover through the autophagy and endolysosomal systems (Di Giovanni and Sheng, 2015; Farfel-Becker et al., 2019; Jin et al., 2018; Roney et al., 2022). Therefore, presynaptic assembly and maintenance require seamless integration of biogenesis, bidirectional transport, and degradation of synaptic components.

Mature presynaptic terminals also contain a local protein synthesis system that transcribes a heterogeneous population of mRNAs supplied by axonal mRNA transport in the form of ribonucleoprotein granules (RNPGs) (Dalla Costa et al., 2021; Hafner et al., 2019). Synaptic mRNA translation has been recognized as a dynamic platform to replenish synapses with new proteins that transduce intrinsic and extrinsic cues into structural and functional presynapse remodeling (Akins et al., 2009). It is less clear whether CAZ proteins can be synthesized locally at nascent presynapses.

The formation and maturation of nascent presynaptic terminals and the remodeling of mature presynapses require tight coordination of the axonal transport of at least seven major cargos and organelles with distinct transport mechanisms: (1) SV-like precursors (SVPs); (2) Piccolo-Bassoon Transport Vesicles (PTVs) containing CAZ scaffolding and membrane proteins; (3) DCVs delivering neuropeptides, (4) receptor tyrosine kinase (RTK)–carrying vesicles and SEs propagating retrograde neurotrophic signaling, (5) mRNAs in the form of RNPGs, (6) mitochondria, and (7) the autophagy and endolysosomal systems (Fig. 1 A). In the next two sections, we provide an overview of axonal transport machineries and mechanisms for targeted delivery of five “primary” presynaptic cargos to ensure the assembly and maintenance of functional presynapses. For axonal transport of mitochondria and autophagy and endolysosomal systems, we refer the readers to recent reviews (Cason and Holzbaur, 2022; Devine and Kittler, 2018; Li and Sheng, 2022; Misgeld and Schwarz, 2017; Roney et al., 2022).

## Axonal transport machineries

Long-distance axonal transport relies on the coordination of three key components of the transport machinery: MTs as trafficking tracks, molecular motors driving cargo transport, and motor adaptors and effectors that selectively connect cargo and motors and/or activate motor processivity.

### MTs

MTs are polarized tubulin polymers with fast-growing plus ends and more stable minus ends. In the axon, parallel MTs form a unipolar array with the plus ends pointing toward the axon terminals. Axonal MTs serve as the major commute tracks for various cargo binding and movement. They are stabilized by MT-associated proteins (MAPs), which can affect motor protein recruitment (Monroy et al., 2018). Various plus-end tracking proteins (+TIPs), such as EB1, accumulate at the growing MT plus end to regulate axonal MT dynamics and facilitate their interactions with motors and cargos (Akhmanova and Steinmetz, 2008; Miryala et al., 2022). Posttranslational MT modifications, including acetylation, detyrosination, and tyrosination, affect cargo trafficking efficiency by regulating motor activity (Kapitein et al., 2010; Sirajuddin et al., 2014; Tas et al., 2017). Tyrosinated tubulin is enriched at the plus ends of growing MTs (near growth cones), while acetylation and detyrosination are more likely to be seen in the middle or minus end of MTs to maintain MT stabilization (Song and Brady, 2015). Kinesin-1 binds preferentially to MTs that are acetylated or detyrosinated to transport cargos along the axon (Hammond et al., 2009; Konishi and Setou, 2009; Nakata et al., 2011), while kinesin-3 and dynein motors prefer to bind to tyrosinated MTs exhibiting non-selective cargo delivery to both axons and dendrites (Nirschl et al., 2016; Tas et al., 2017). However, the mechanisms for these modifications in selectively guiding motor-MT engagement within axons versus dendrites remain largely unknown.

### Motor proteins

Kinesin and dynein are two major classes of MT-based and ATP-driven molecular motors that move cargos in the anterograde (from the soma to distal axons) and retrograde (from axonal terminals to the soma) directions, respectively. The kinesin superfamily (KIF) constitutes at least 45 genes in the human genome, 38 of which are expressed in brain, and is classified into 15 subfamilies, designated as kinesin-1 to kinesin-14B (Hirokawa et al., 2010). Motors from four of these subfamilies—kinesin-1 (KIF5A, KIF5B, and KIF5C), kinesin-2 (KIF3 and KIF17), kinesin-3 (KIF1A, KIF1B, KIF13, and KIF16B), and kinesin-4 (KIF21A)—drive long-distance trafficking of diverse cargos and organelles from the soma into the axon (Gicking et al., 2022). Kinesin-1 motors are formed from two heavy chains (KHCs) and two light chains (KLCs); each KHC contains a catalytic motor domain that binds to MTs and generates movement via ATP hydrolysis, a neck linker, a coiled-coil stalk domain that mediates dimerization, and an inter-head to a tail domain that associates with a KLC. Kinesin-1 mediates axonal transport of AZ precursors PTVs, mitochondria, and RNA granules (Fukuda et al., 2021; Pilling et al., 2006; Su et al., 2004). Kinesin-2 motors drive the anterograde motility of vesicles containing presynaptic sCAM proteins, including N-cadherin and β-catenin (Teng et al., 2005). Kinesin-3 motors drive the motility of SVPs, DCVs, SEs, and RNA granules (Bentley et al., 2015; Lo et al., 2011; Okada et al., 1995; Pichon et al., 2021).

In contrast to such diversity in kinesin motor subfamilies, retrograde axonal transport is mediated by a single cytoplasmic dynein motor, which is a large complex consisting of two heavy chains (DHCs) with ATPase activity, two intermediate chains (DICs), two light intermediate chains (DLICs), and several light chains (DLCs) (Reck-Peterson et al., 2018). In addition, the dynactin complex interacts with dynein and is required for motor-cargo association and motor processivity (Gill et al., 1991; King and Schroer, 2000). The actin-based myosin motors, including myosins V and VI, drive short-range cargo trafficking along actin filaments (F-actin). At presynaptic terminals, F-actin is highly enriched and serves as a platform for CAZ assembly, SV recruitment and recycling, and the anchor of presynaptic mitochondria, thus controlling presynaptic cargo switch from MT-based trafficking to actin-based anchoring (Cingolani and Goda, 2008; Gutnick et al., 2019; Li et al., 2020; Schroeder et al., 2010).

### Motor adaptors and effectors

A diverse array of adaptors and effectors have been identified to serve as a linker recruiting motor proteins to specific cargos/organelles or act as effectors activating the motility of kinesin or dynein motors. These include the kinesin adaptors liprin-α, Arl8, syntabulin, JIP1, JIP3, and Huntingtin (HTT). All of these link either the kinesin KHC tail or KLC to its trafficking cargo and/or activate the motility of anterograde axonal transport (Colin et al., 2008; Klassen et al., 2010; Miller et al., 2005; Su et al., 2004; Verhey et al., 2001). For dynein motors, adaptors/effectors include HTT, Huntingtin-associated protein-1 (HAP1), BICD1 and 2 (N-terminal domain of protein bicaudal D homolog 1 and 2), Hook1 and 3, Snapin, and JIP3. These proteins connect specific cargos with dynein motors, enhance dynein–dynactin interaction, or activate retrograde processivity (Caviston et al., 2007; Celestino et al., 2022; Engelender et al., 1997; Olenick et al., 2016; Schlager et al., 2014; Zhou et al., 2012).

## Presynaptic cargos and their transport mechanisms

### SVPs

Mature SVs consist of >130 proteins involved in SV exocytosis and endocytosis, trafficking, and refilling of neurotransmitters (Blondeau et al., 2004; Morciano et al., 2005; Takamori et al., 2006; Taoufiq et al., 2020). These SV components are transported separately in distinct SVP forms and then assembled through a recycling endosome trafficking route. An immuno-EM study in developing rat hippocampal neurons revealed pleiomorphic vesicles (50–300 nm in size) carrying SV integral membrane proteins, while SV-associated proteins synapsin and α-synuclein proceed through different routes of biosynthesis and axon transport and are sorted into the same SV clusters when they are in axons (Tao-Cheng, 2020). Upon transport into nascent presynapses, SVPs are thought to form mature SVs by undergoing constitutive exo-endocytic cycling locally or by directly budding from SVPs (Rizalar et al., 2021).

**Figure 2.**
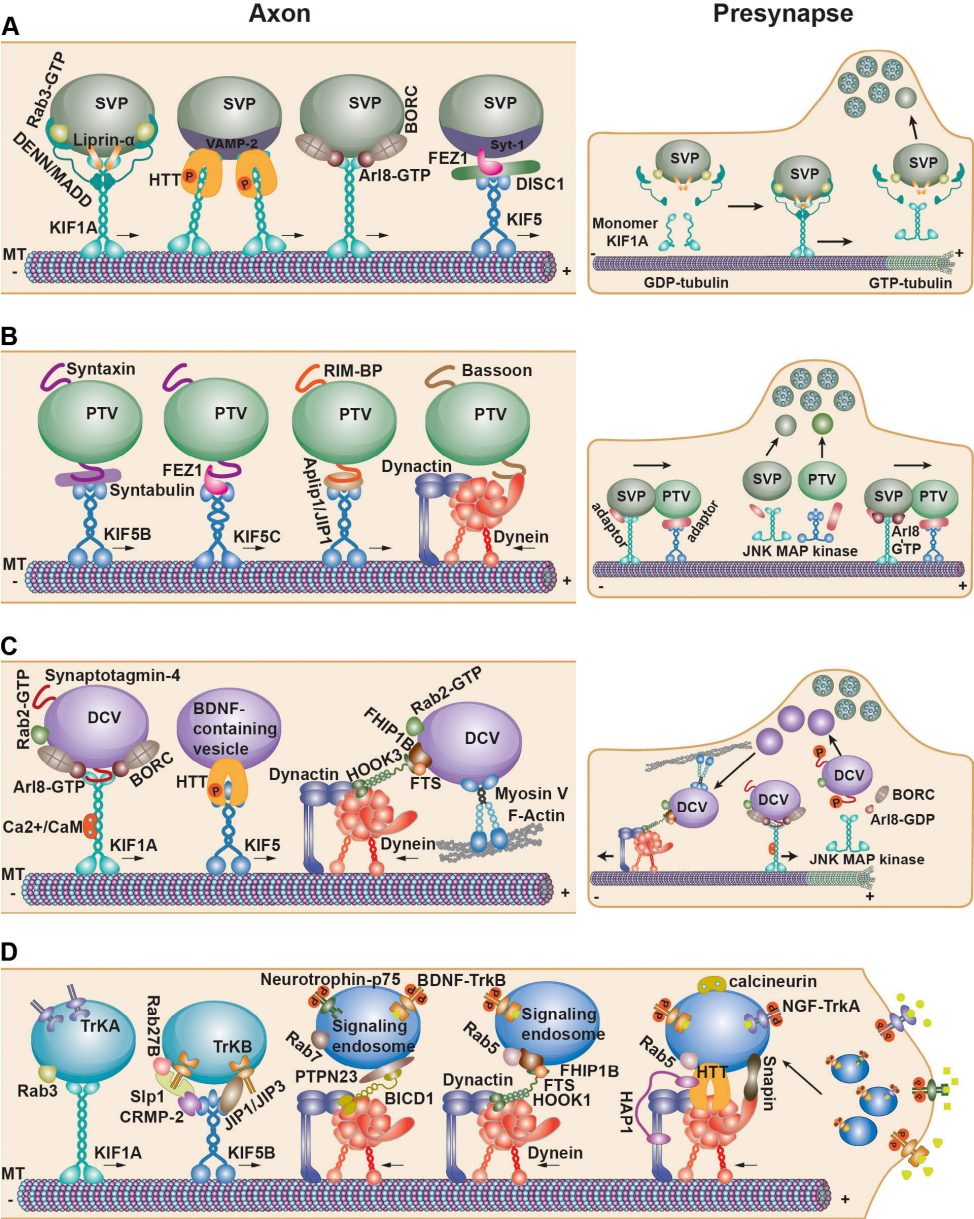
**Mechanisms driving presynaptic cargo transport. (A)** The SVP transport machinery. Anterograde SVP transport is driven by the kinesin motor KIF1A, which is autoinhibited by the binding of its stalk coiled-coil domain to its motor heads. Cargo specificity is achieved by adaptor/effector proteins which bind to the stalk domain to relieve motor autoinhibition. DENN/MADD binds to the KIF1A stalk region and recruits the motor to SVP membranes through cooperation with Rab3-GTP. Liprin-α and HTT serve as adaptors to recruit KIF1A to SVPs. KIF1A is also relieved from its autoinhibition status by cargo-mediated dimerization, resulting in increased binding and processivity on MTs compared with its monomeric state. The BORC–Arl8–KIF1A complex also drives SVP transport into axons. In addition, KIF5 motors drive axonal transport of Syt-1–carrying SVPs through interactions with FEZ1-DISC1. Upon arrival at *en passant* presynaptic boutons, SVPs are unloaded by detaching motors from the GTP-tubulin bound MT plus ends. **(B)** The PTV transport machinery. PTVs are distinct 80-nm dense core vesicles containing the CAZ scaffolds (Piccolo and Bassoon), CAZ proteins (Munc13-1, RIM1α, and ELKS2), sCAM N-cadherin, and presynaptic plasma membrane proteins Syntaxin-1 and SNAP-25. Syntabulin functions as a KIF5 adaptor driving PTV transport via an interaction with syntaxin. Syntabulin loss-of-function leads to impaired axonal transport of PTVs from the soma toward distal axons. FEZ1 binds syntaxin, thus loading KIF5C onto PTVs. In addition, Aplip1/JIP1 act as a kinesin-1 adaptor to mediate RIM-BP–carrying PTV transport into presynapses. Dynein motors drive retrograde PTV transport by binding to Bassoon. PTVs are cotransported with SVPs facilitated by Arl8-GTP bound to SVP membrane. The JNK-MAP kinase pathway suppresses anterograde transport and promotes SV and AZ protein clustering for presynaptic assembly. **(C)** The DCV transport machinery. DCVs convey neuropeptides and neurotrophins along axons to modulate synapse function. Both KIF1A and KIF5 drive anterograde DCV transport into axon terminals, while dynein/dynactin mediates DCV retrograde transport. Rab2 is recruited to DCVs and assists the BORC-Arl8-KIF1A complex in mediating axonal transport, which is also regulated by Ca^2+^/calmodulin upon increased neuronal activity. Phosphorylated HTT promotes anterograde transport of BDNF-containing DCVs by increasing kinesin-1 recruitment. Rab2 also helps recruit dynein to DCVs, and HOOK3 activates dynein–dynactin motility. Myosin Va binds to DCVs via its tail domain and facilitates retrograde transport of DCVs. Phosphorylation of Syt-4 (S135) on DCV membranes by JNK MAP kinase destabilizes Syt4-KIF1A coupling and thus unloads DCVs at *en passant* presynaptic boutons. **(D)** The SE transport machinery. KIF1A is the motor driving anterograde transport of TrkA in Rab3-positive secretory vesicles to axon terminals. Anterograde transport of TrkB is driven by the Slp1–CRMP-2–KIF5B complex in a Rab27B-dependent manner. JIP3 also binds TrkB and mediates TrkB anterograde transport by linking TrkB and KLC. At presynaptic terminals, Rab5-positive SEs are formed following neurotrophin binding to its receptors and undergo retrograde transport toward the soma. Dephosphorylated HTT by calcineurin in response to elevated Ca^2+^ leads to kinesin-1 detachment and facilitates retrograde transport of SEs carrying BDNF-TrkB by dynein motors. HAP1 acts a dynein activator. Hook1 acts as a dynein effector to drive retrograde transport via forming the FTS–Hook–FHIP1B–Rab5 complex. Other adaptor proteins, such as BICD1, PTPN23, and Snapin, also recruit dynein motors to SEs to drive their retrograde signaling.

Anterograde SVP transport is driven by kinesin-3 motors (Fig. 2 A), including UNC-104 in *C. elegans*, Imac in *Drosophila*, and KIF1A and KIF1Bβ in mammals and humans (Hall and Hedgecock, 1991; Pack-Chung et al., 2007; Okada et al., 1995; Zhao et al., 2001). Kinesin-3 is a monomeric motor that is autoinhibited by the binding of its stalk coiled-coil domain to its motor heads. Relief from autoinhibition is achieved by cargo-mediated motor dimerization, which promotes the fast anterograde transport of SVPs (>3 μm/sec) with a few pauses along GDP-rich MT lattices (Hammond et al., 2009; Tomishige et al., 2002). However, at MT plus ends at presynaptic terminals, where GTP-tubulin is enriched, KIF1A shows weak MT-binding affinity, thus favoring SVP release upon arrival at *en passant* synapses (Guedes-Dias et al., 2019) (Fig. 2 A). In *C. elegans*, null mutants of *unc-104* lead to impaired axonal transport of SVPs, reduced number of SVs at presynapses, and deficits in locomotion (Hall and Hedgecock, 1991). Reduced UNC-104 levels in *unc-104*^+/−^ cause SVP pausing at branch points, impeding their entry into synaptic terminals (Vasudevan et al., 2024). In *Drosophila*, *Imac* knockout impairs the axonal transport of SVPs and formation of presynaptic boutons (Pack-Chung et al., 2007). In mice, a *KIF1A* loss-of-function mutation leads to decreased axonal transport of SVPs and their accumulation in the soma, along with a dramatic reduction of mature SVs at synapses, which are associated with sensorimotor deficits and early postnatal death (Yonekawa et al., 1998). Conversely, KIF1A overexpression promotes presynaptic bouton formation (Kondo et al., 2012). In humans, a set of mutations in *KIF1A* and *KIF1Bβ* have been linked to abnormal axonal transport of SVPs and synapse phenotypes observed in NDDs (Chiba et al., 2023).

Kinesin-3 motors possess a C-terminal pleckstrin homology (PH) domain and a conserved stalk domain. The PH domain binds phosphatidylinositol-4,5-bisphosphate on the cargo membrane, which is critical for SVP loading onto motors (Klopfenstein and Vale, 2004). Cargo specificity is achieved by adaptors, which bind to the stalk domain (Fig. 2 A). For example, liprin-α interacts directly with KIF1A through its coiled-coil domain (Shin et al., 2003); liprin-α mutations decrease anterograde SVP transport (Miller et al., 2005). Similarly, HTT acts as a scaffolding protein that colocalizes with KIF1A on VAMP2-positive SVPs. HTT phosphorylation at S421 recruits KIF1A to SVPs and thus enhances SVP transport (Vitet et al., 2023). The death domain of DENN/MADD (differentially expressed in normal and neoplastic cells/MAP kinase activating death domain), a guanine nucleotide exchange factor, binds to the stalk region of KIF1A and KIF1Bβ, while the MADD domain interacts with the GTPase-Rab3 on SVP membranes (Niwa et al., 2008). Thus, DENN/MADD connects kinesin-3 with SVPs carrying GTP-bound Rab3. RAB3A phosphorylation disrupts its binding to MADD, thus preventing SVP loading onto KIF1A/KIF1Bβ motors for anterograde transport (Dou et al., 2024). In *DENN/MADD* knockout mice, SV numbers are reduced (Tanaka et al., 2001). In *C. elegans*, the GTPase Arl8 activates unc-104/KIF1A by relieving autoinhibition in a GTP-dependent manner (Niwa et al., 2016). *ARL8* loss-of-function results in SV accumulation at proximal axons and a loss of distal boutons due to insufficient unc-104/KIF1A activation (Klassen et al., 2010). Therefore, this GTPase switch may regulate SVP transport into presynaptic terminals. Fasciculation and elongation protein zeta-1 (FEZ1) acts as an adaptor that activates kinesin-1 and thus drives the axonal transport of Synaptotagmin-1 (Syt-1)–carrying SVPs (Blasius et al., 2007). The cargo-motor coupling is controlled by the phosphorylation state of FEZ1 via the kinase UNC-51 (Toda et al., 2008). Interestingly, Disrupted in Schizophrenia-1 (DISC1), a genetic risk factor for SZ, regulates Syt-1–carrying SVP transport by binding both kinesin-1 and FEZ1; *DISC1* mutation causes defective transport by disrupting motor–cargo assembly (Flores et al., 2011).

### PTVs

The CAZ is organized by a set of multidomain proteins, including two large scaffolding proteins Piccolo and Bassoon, ELKS/CAST, Munc13s, Rab3-interacting molecules (RIMs), RIM-binding proteins (RIM-BPs), and liprin-α (Südhof, 2012). After being synthesized in the soma, the CAZ components are sorted and assembled into distinct sets of Golgi-derived transport cargos termed PTVs (Ackermann et al., 2015; Garner et al., 2000). PTVs are 80-nm dense core vesicles containing CAZ scaffolds, CAMs N-cadherin, and presynaptic plasma membrane proteins Syntaxin and SNAP-25. PTVs also gather Munc13-1 and RIM1α in a post-Golgi step forming a “mature” PTV (Maas et al., 2012). The CAZ functions to dock and prime SVs for exocytosis, recruit VGCCs to release sites, and tether synaptic adhesion molecules. Individual CAZ scaffold size and composition can scale synaptic strength by affecting SV release probability (Holderith et al., 2012). As few as five PTVs could provide sufficient scaffold proteins to form a functional CAZ in developing neurons. PTVs have been suggested to appear prior to SVP arrival at nascent boutons (Ahmari et al., 2000; Friedman et al., 2000; Zhai et al., 2000) or are cotransported with SVPs during development (Tao-Cheng, 2020; Vukoja et al., 2018). The SNARE proteins syntaxin 1 and SNAP-25 are also cotransported with Piccolo and Bassoon, indicating that the SV fusion machinery is packaged with the CAZ to lay the structural foundation for recruiting other presynaptic components and SVs (Zhai et al., 2001). Similarly, VGCCs are thought to be assembled in the soma and cotransported to presynapses (Macabuag and Dolphin, 2015). Therefore, multiple presynaptic proteins are cotransported by the same precursor organelle or by clusters of “distinct” carriers through a coordinated transport that ensures the delivery of stoichiometric amounts of AZ and SV contents for efficient presynaptic assembly in developing neurons (Bury and Sabo, 2011; Wu et al., 2013). However, the current concept of a “distinct” presynaptic carrier model requires further validation concerning their molecular identity, biogenesis sorting, and transport mechanisms.

Kinesin-1 motors KIF5B and KIF5C drive anterograde PTV transport (Fig. 2 B). Syntabulin functions as a kinesin-1 adaptor driving PTV transport via an interaction with PTV-carrying cargo syntaxin 1 (Su et al., 2004). Syntabulin loss-of-function leads to impaired transport of PTVs from the soma toward distal axons and reduced density of presynapses and AZs in developing neurons and mature CNS in mice (Cai et al., 2007; Xiong et al., 2021). The second adaptor-like protein for driving PTVs is FEZ1, which binds syntaxin 1 and Munc-18 and thus loads KIF5C onto the syntaxin-containing PTVs (Chua et al., 2012). APP-like protein-interacting protein 1 (Aplip1), a homolog of JIP1 functioning as a kinesin-1 adaptor, interacts with RIM-BP through its proline-rich (PxxP) motif. The mutation in the motif leads to ectopic accumulation of RIM-BP–enriched PTVs due to defective anterograde transport (Siebert et al., 2015). PTVs have been reported to cotransport with SVPs, promoting SVP clustering and capture (Bury and Sabo, 2011; Lipton et al., 2018; Vukoja et al., 2018). As a motor adaptor, Arl8 facilitates kinesin-cargo coupling and promotes anterograde cotrafficking of PTVs and SVPs (Wu et al., 2013). The JNK-MAP kinase signaling pathway promotes SV and AZ protein clustering for presynaptic assembly. Interestingly, Bassoon itself functions as an adaptor binding DLC for retrograde PTV transport. Disruption of Bassoon–DLC interactions impairs trafficking and distribution of Piccolo and Bassoon along axons (Fejtova et al., 2009), providing insights as to how bidirectional PTV transport is critical for precise targeting of presynaptic scaffolds at *en passant* synaptic sites.

### DCVs

DCVs convey neuropeptides and neurotrophins along axons to modulate synaptic maturation and plasticity in a neuron-type-dependent manner (Altar et al., 1997; van den Pol, 2012). Axonal DCV transport displays a complex motility pattern: long-range circulation and sporadic capture (Bharat et al., 2017; Wong et al., 2012). The small GTPase Rab2 mediates DCV biogenesis and maturation in the cell body (Ailion et al., 2014; Edwards et al., 2009). Either kinesin-3 alone or kinesin-3 and kinesin-1 work in concert to drive anterograde transport of DCVs from the soma into axons (Lim et al., 2017; Lo et al., 2011), while the dynein/dynactin complex mediates DCV retrograde transport (Kwinter et al., 2009) (Fig. 2 C). The BORC subunit Blos1 activates Arl8 to a GTP-bound state to promote its binding and activation of KIF1A, thus driving DCV transport into axons. Rab2 is recruited to DCVs by Ema, thus assisting the BORC–Arl8–kinesin complex in driving the axonal transport of DCVs (Lund et al., 2021). Anterograde transport of DCVs slows down and frequently pauses at and near presynapses (Guedes-Dias et al., 2019; Nassal et al., 2022), where DCVs are preferentially captured in an activity-dependent manner. The calcium-binding protein calmodulin (CaM) binds to KIF1A in response to Ca^2+^ signaling and thus facilitates DCV trafficking upon increased neuronal activity (Cavolo et al., 2016; Stucchi et al., 2018). KIF1Bβ contains a conserved CaM binding site and likely undergoes a similar Ca^2+^/CaM-dependent regulation of DCV transport. In addition, HTT phosphorylation by AKT promotes anterograde transport of brain-derived growth factor (BDNF)–containing DVCs by increasing kinesin-1 recruitment (Colin et al., 2008).

Syt-4 binds KIF1A and is cotransported with DCVs. Phosphorylation of Syt-4 (S135) by JNK kinase, which is upregulated upon neuronal activity, destabilizes Syt4-KIF1A coupling, leading to a transition from MT-based DCV trafficking to F-actin-based capture at *en passant* presynaptic boutons (Bharat et al., 2017). The Syt4-KIF1A coupling is also modulated by Ca^2+^/CaM binding to KIF1A (Stucchi et al., 2018). DCV capture at synapses may also rely on liprin-α, which captures KIF1A-bound DCVs upon their synapse entry by interacting with KIF1A (Goodwin and Juo, 2013). At presynaptic boutons, uncaptured DCVs can be converted to a retrograde transport mode and return to the proximal axon, where they again switch to the anterograde transport route (Wong et al., 2012). Rab2 recruits dynein to DCVs with the help of HOOK3 to activate dynein–dynactin motility (Lund et al., 2021; Olenick et al., 2016). Myosin Va binds to DCVs via its tail domain and facilitates retrograde DCV axonal transport (Bittins et al., 2010). Therefore, fine-tuned coordination of anterograde versus retrograde DCV motility contributes to presynaptic targeting of neuropeptides and neurotrophins.

### SEs

Propagation of retrograde neurotrophic signaling conveyed by SEs is essential for axon growth, synaptogenesis, and plasticity. At presynapses, neurotrophins, including nerve growth factor, BDNF, neurotrophin-3 or -4/5, bind RTKs of the Trk family or the p75 neurotrophin receptor (p75^NTR^) to trigger the internalization of ligand-bound receptors into Rab5-positive SEs (Chao, 2003). Trk-harboring SEs can function locally by promoting axonal growth and synapse formation, or distally by transporting to the soma to activate transcriptional signaling necessary for synapse maturation and plasticity (Harrington and Ginty, 2013). To trigger retrograde neurotrophic signaling, the prerequisite is the trafficking of newly synthesized RTKs (TrkA/TrkB) from the soma to axon tips. Kinesin-1 (KIF5B) and kinesin-3 (KIF1A) motors drive anterograde transport of RTKs in Rab3- or Rab27B-positive secretory vesicles (Fig. 2 D) (Scott-Solomon and Kuruvilla, 2018). In mouse sensory neurons, anterograde transport of TrkA-carrying cargos is driven by KIF1A with the help of GTP-bound Rab3. Dorsal root ganglia from *Kif1a*^+/−^ mice exhibit progressive sensory neuron loss and sensory neuropathy (Tanaka et al., 2016). TrkB-carrying cargos are loaded onto KIF5B by a complex of CRMP-2, Slp1, and Rab27B. The cytoplasmic tail of TrkB binds to Slp1 in a Rab27B-dependent manner, and CRMP-2 connects Slp1 to KIF5B (Arimura et al., 2009). JIP3 also mediates TrkB anterograde transport by linking TrkB and KLC in mouse hippocampal neurons (Huang et al., 2011). JIP1 and JIP3 form a complex that functions to relieve kinesin-1 autoinhibition (Sun et al., 2017); overexpressing JIP1 or JIP3 enhances TrkB–KLC interaction and promotes TrkB transport, while knocking down JIP1 or JIP3 diminishes TrkB anterograde transport. It was also reported that Rab6-positive TrkB carriers are driven by KIF1A for anterograde transport in hippocampal neurons (Zahavi et al., 2021).

After internalization at presynaptic terminals, Trk and its ligands—neurotrophins—are sorted into Rab5-positive early endosomes and then trafficked to Rab7-positive late endosomes targeted for dynein-driven retrograde transport toward the soma (Fig. 2 D) (Deinhardt et al., 2006; Heerssen et al., 2004). In hippocampal neurons, BDNF-TrkB signaling activates PI3K that promotes anterograde transport of TrkB cargos into the nascent axon and further enhances surface insertion of TrkB, creating a self-amplifying feed-forward loop to promote axon growth (Cheng et al., 2011). HTT, as a BDNF scaffold, can be dephosphorylated by calcineurin in response to Ca^2+^ transients in synapses (Scaramuzzino et al., 2022). Dephosphorylation of HTT (S421) leads to kinesin-1 detachment and thus promotes retrograde transport of SEs carrying BDNF-TrkB by binding to dynein (Colin et al., 2008).

Neurotrophin signaling mediated by SEs conveys synapse-to-nucleus communication, helping neurons respond to presynaptic signaling with transcriptional changes (Terenzio et al., 2017; Yamashita, 2019). Multiple motor adaptors/effectors and sorting pathways are involved in the retrograde transport of SEs. HAP1 is required for TrkB internalization upon BDNF binding and activation of dynein motility through its interaction with the p150^Glued^ dynactin subunit (Lim et al., 2018). Hook1 acts as a dynein effector to drive retrograde transport of both Rab5- and Rab7-positive SEs carrying BDNF-TrkB (Olenick et al., 2019), likely by forming an FTS–Hook–FHIP1B–Rab5 complex (Christensen et al., 2021) (Fig. 2 D). BICD1, a dynein adaptor, is also necessary for retrograde transport of TrkB- and p75-containing SEs (Terenzio et al., 2014). PTPN23, a member of the endosomal sorting complex, binds BICD1 and contributes to dynein recruitment to SEs (Budzinska et al., 2020). Snapin, an adaptor binding to dynein DIC, helps recruit dynein motors to BDNF-TrkB–carrying SEs for retrograde axonal transport (Zhou et al., 2012). *Snapin* knockout mice exhibit embryonic and neonatal death accompanied by abnormal brain development manifested as reduced cortical plates and cell density (Zhou et al., 2011). Some activated BDNF-TrkB complexes are colocalized with LC3b-II–positive autophagic organelles that undergo retrograde transport to confer long-range signaling capabilities (Kononenko et al., 2017; Andres-Alonso et al., 2019). Upon reaching the soma, SEs exhibit extended periods of signal up to 25 h via Coronin-1–mediated local recycling and reinternalization of RTKs into Rab11-positive recycling endosomes that escape lysosomal targeting and degradation (Moya-Alvarado et al., 2022; Suo et al., 2014). Rab11-positive endosomes can further traffic recycled RTKs outward to the axon for a feedback loop of neurotrophic signaling amplification (Ascaño et al., 2009). BICD1 also plays a role in maintaining the balance between RTK receptor degradation and recycling (Terenzio et al., 2014). Such persistent singling may help activate transcriptional programs necessary for neurite growth and synapse formation. SEs originating from distal axons can also be transported all the way to dendrite arbors to regulate synaptic connectivity (Sharma et al., 2010).

### mRNA transport via RNA granules

One mechanism of presynaptic protein replenishment involves the axonal transport of mRNAs in RNA granules to presynaptic sites for local translation (Batista et al., 2017; Turner-Bridger et al., 2018). RNA profiling studies have revealed more than 1,000 different mRNAs enriched in axons and presynaptic terminals, thus providing a platform for local protein synthesis for structural and functional maintenance and remodeling of presynapses (Cajigas et al., 2012; Dalla Costa et al., 2021; Hafner et al., 2019). In neurons, mRNAs transcribed in the nucleus are packaged into large protein complexes called ribonucleoproteins (RNPs) through association with RNA-binding proteins (RBPs). After nuclear export, RNPs undergo long-distance axonal transport driven directly by motors (Kanai et al., 2004; Sladewski et al., 2013). mRNA transport in axons may also occur via hitchhiking of RNA granules on moving organelles, such as endosomes, lysosomes, or mitochondria (Cioni et al., 2019; Gershoni-Emek et al., 2018; Liao et al., 2019). Synaptic mRNA arrest and anchoring are thought to rely on F-actin as well as deactivation of driving motors (Sladewski et al., 2013).

The 3′ and 5′ untranslated regions of mRNAs play key roles in transport and localization selectivity (Andreassi and Riccio, 2009; Merianda et al., 2013; Tushev et al., 2018). Multiple motifs or *cis*-acting elements are necessary and sufficient for mRNA localization into axons, including the “zip code” motif, MAIL (mail for axonal importin localization) motif, and AU-rich sequences bearing AUUUA element; many RBPs can also bind to a single RNA element, suggesting that different mRNAs may be cotransported by the same RBP in a single RNA granule (Lee et al., 2018). The number of localization motifs on a single mRNA is linearly correlated with the number of motors loaded, thus affecting its processivity or run length (Sladewski et al., 2013). However, how different RBPs recruit motor proteins to form a transport granule in axons and whether this process requires additional adaptors remains unclear. Future studies are needed to reveal mechanisms underlying localization and translation of mRNA for the maintenance of synaptic remodeling and plasticity.

## NDD-linked mutations in the axonal transport machinery

During nervous system development, any perturbation in axonal transport will cause a mistargeted distribution of presynaptic proteins/cargos, leading to impaired presynaptic formation and maintenance. Missense mutations and small genomic deletions or rearrangements in genes encoding the transport machinery are increasingly revealed via disease-related genome screening. Growing lines of evidence indicate that axonal transport disruption contributes to a broad spectrum of NDDs. Presynaptic assembly and maturation are regulated at multiple levels and through redundant mechanisms that integrate biosynthesis, transport, and assembly of presynaptic building blocks (Emperador-Melero and Kaeser, 2020). It is thus challenging to determine whether defective axonal transport alone has a major pathogenic role or is just an epiphenomenon that may occur in a short time window. In this section, we summarize NDD-associated mutations in genes encoding transport machineries that result in a wide range of NDD phenotypes, including lissencephaly, ASD, mental retardation, ID, cognitive and motor impairment, complex cortical malformations, and infant-onset epilepsy (Table 1). Although some of these mutations impair MT stability, motor activity, or motor-cargo coupling, presynaptic assembly and maintenance have not been examined *in vitro* or *in vivo* nerve systems. One of the well-characterized models with identifiable phenotypes of defective axonal transport and presynaptic assembly is the ASD-linked mutation of KIF5 adaptor syntabulin for PTV transport (Xiong et al., 2021). These observations may direct future studies by testing the emerging hypothesis that defective axonal transport is one of the mechanisms contributing to synaptic pathology in NDDs (Badal and Puthanveettil, 2022; Lasser et al., 2018; Sleigh et al., 2019).

**Table 1.** NDD-linked mutations in genes encoding axonal transport machineries

Protein	Gene with mutation	Inheritance	Disease: Phenotypes	References
Microtubules				
α1A-tubulin	*TUBA1A*	AD: Missense/insertion/deletion mutations	LIS3: Congenital microcephaly, mental retardation, no language development	Fallet-Bianco et al., 2014; Poirier et al., 2013
β2A-tubulin Class IIa	*TUBB2A*	AD: Missense mutations	CDCBM5: Intellectual disability, hypotonia, developmental delay, epilepsy	Cushion et al., 2014; Rodan et al., 2017; Schmidt et al., 2021
β-tubulin Class I	*TUBB*	AD: Missense mutations	CDCBM6: Delayed psychomotor development and microcephaly	Breuss et al., 2012
β2B-tubulin Class IIb	*TUBB2B*	AD: Missense mutations	CDCBM7: Microcephaly, mental retardation, severe neuromotor impairment, no visual contact, infantile seizures	Cederquist et al., 2012; Laquerriere et al., 2016
β3-tubulin Class III	*TUBB3*	AD: Missense mutations	CDCBM1: Mental retardation, strabismus, axial hypotonia, and spasticity	Fallet-Bianco et al., 2014; Poirier et al., 2010
β6-tubulin Class V	*TUBB6*	AD: Missense mutations	FPVEPD: Bilateral ptosis and facial palsy, severe rhinophonia aperta with speech articulation defects	Fazeli et al., 2017
γ1-tubulin	*TUBG1*	AD: Missense mutations	CDCBM4: Complex cortical malformations, intellectual disability	Poirier et al., 2013
Motor proteins				
Kinesin-1	*KIF5A*	AD: Missense mutations in tail domain	NEIMY: Myoclonic seizures, lack of developmental progress	Duis et al., 2016; Rydzanicz et al., 2017
		AD: Missense mutations in motor/stalk/tail domain	SPG 10: Limb spasticity and weakness, sensorimotor polyneuropathy, cognitive impairment	de Boer et al., 2021; de Souza et al., 2017; Méreaux et al., 2022
		AD: Missense mutations in stalk domain	CMT2: Chronic axonal motor and sensory polyneuropathy	de Boer et al., 2021
	*KIF5B*	AR: Missense mutations	Developmental and speech delay	Charng et al., 2016
	*KIF5C*	AD: Missense mutations in motor domain	CDCBM2: Microcephaly, developed clonic seizures, severe intellectual disability	de Ligt et al., 2012; Poirier et al., 2013
Kinesin-2	*KIF3B*	AD: Missense mutations in motor domain	SZ: Cognitive impairment, delusions, hallucinations, disorganized speech and movements	Alsabban et al., 2020
Kinesin-3	*KIF1A*	AD: Missense mutations in motor domain	NESCAVS: Global developmental delay, intellectual disabilities, seizures	Lee et al., 2015; Ohba et al., 2015
		AD: Missense mutations in motor domain	HSES: Severe brain edema and atrophy, developmental delay, peripheral neuropathy, autonomic dysfunction	Isobe et al., 2022
		AR: Truncating mutation	HSN2C: Early onset of hereditary sensory neuropathy, distal muscle weakness, slowed speech development	Rivière et al., 2011
		AD/AR: Missense mutations in motor domain	SPG30: Early childhood onset unsteady spastic gait, hyperreflexia of the lower limbs, learning disabilities	Citterio et al., 2015; Klebe et al., 2012; Nemani et al., 2020; Pennings et al., 2020
	*KIF1Bβ*	AD: Missense mutations in motor domain	CMT2A: Chronic axonal motor and sensory polyneuropathy	Xu et al., 2018; Zhao et al., 2001
Dynein	*DYNC1H1*	AD: Missense mutations in motor/tail domain	CDCBM13: Global developmental delay, intellectual disability, seizures	Poirier et al., 2013; Vissers et al., 2010; Willemsen et al., 2012
		AD: Missense mutations in tail domain	SMALED1: Early childhood onset of muscle weakness and atrophy	Harms et al., 2012; Tsurusaki et al., 2012
Myosin V	*MYO5A*	AR: Nonsense or truncating mutation	Hindbrain malformation and developmental delay	Charng et al., 2016
Motor adaptors and regulatory proteins				
Kinesin-binding protein	*KIF1BP/KBP*	AR: Nonsense mutation	GOSHS: Intellectual disability, microcephaly	Brooks et al., 2005; Drévillon et al., 2013
		AR: Truncating mutations	Polymicrogyria: Microcephaly	Valence et al., 2013
Bicaudal D2	*BICD2*	AD: Missense mutations	SMALED2A: Early childhood onset of muscle weakness and atrophy	Neveling et al., 2013; Peeters et al., 2013
		AD: Missense mutations	SMALED2B: Decreased fetal movements, severe hypotonia, muscle atrophy, and respiratory insufficiency after birth	Koboldt et al., 2018; Ravenscroft et al., 2016; Storbeck et al., 2017
Lissencephaly 1	*PAFAH1B1*	AD: Missense or truncating mutations	LIS: Developmental delay, early onset of seizures	Reiner et al., 1993; Saillour et al., 2009
NudE neurodevelopment protein 1	*NDE1*	AR: Truncating or nonsense mutations	LIS4: Severe microcephaly, mental retardation, early-onset epilepsy	Abdel-Hamid et al., 2019; Alkuraya et al., 2011
		AR: Nonsense mutation or intragenic deletion	MHAC: Microcephaly, motor and mental retardation	Abdel-Hamid et al., 2019; Guven et al., 2012
Syntabulin	*SYBU*	N/A: Missense mutation	Autism: Delayed or absent language development, learning disability, repetitive speech or motor behaviors, social deficits, seizure	Herman et al., 2016; Xiong et al., 2021
Disrupted in schizophrenia 1	*DISC1*	N/A: Missense mutation	SZ9: Cognitive impairment, delusions, hallucinations, disorganized speech and movements	Schumacher et al., 2009; Song et al., 2008
FMRP	*FMR1*	XLD: Missense and truncating mutations, unstable expanded CCG repeat	FXS: Impaired intellectual development, autistic traits, distinct facial features, and seizures	De Boulle et al., 1993; Grønskov et al., 2011; Kremer et al., 1991
CLIPs	*CLIP1*	AR	ARID: Intellectual disability	Larti et al., 2015

NDD-linked genetic mutations in genes encoding the axonal transport machinery, along with their inheritance patterns and disease phenotypes.

Inheritance: AD, autosomal dominant; AR, autosomal recessive; N/A, not applicable; XLD, X-linked dominant; ARID, autosomal recessive intellectual disability; CDCBM1-13, complex cortical dysplasia with other brain malformations-1-13; CMT2, Charcot-Marie-Tooth disease 2; FPVEPD, facial palsy with ptosis and velopharyngeal dysfunction; FXS, fragile X syndrome; GOSHS, Goldberg-Shprintzen syndrome; HSES, hemorrhagic shock and encephalopathy syndrome; HSN2C, hereditary sensory neuropathy type IIC; IAHSP, infantile-onset ascending hereditary spastic paralysis; LIS, lissencephaly; LIS3, lissencephaly 3; LIS4, lissencephaly 4; MHAC, microhydranencephaly; NEIMY, neonatal intractable myoclonus; NESCAVS, neurodegeneration and spasticity with or without cerebellar atrophy or cortical visual impairment syndrome; SCZD, schizophrenia; SMALED1, lower extremity-predominant spinal muscular atrophy-1; SMALED2A, childhood-onset lower extremity-predominant spinal muscular atrophy-2A; SMALED2B, prenatal-onset lower extremity-predominant spinal muscular atrophy-2B; SPG10, spastic paraplegia type 10; SPG30, spastic paraplegia type 30.

### Microtubules

**Figure 3.**
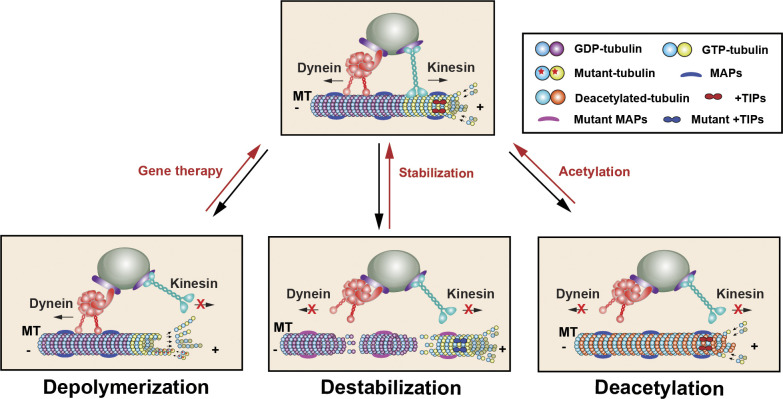
**NDD-linked mutations in MTs.** MTs are hollow tubes that are composed of α- and β-tubulin heterodimers, which undergo dynamic cycles of growth (polymerization) and shrinkage (depolymerization). Newly incorporated tubulin is bound to guanosine triphosphate (GTP) that gets rapidly hydrolyzed upon polymerization, generating guanosine diphosphate (GDP)–bound tubulin along the MT lattice. Hundreds of mutations in genes encoding tubulin were reported in patients presenting with NDDs. These mutations were thought to disrupt MT dynamics via switching from growth to shrinkage (depolymerization), which disrupts axon growth, presynaptic cargo transport, and synaptogenesis. MT dynamics are also regulated by MAPs and +TIPs. Mutations in these proteins were reported in patients with NDDs, resulting in MT destabilization and impaired axonal transport. MT-stabilizing agents have shown some beneficial effects in ameliorating neurological and behavioral deficits in some models of NDDs. The C-terminal tail of α-tubulin undergoes posttranslational modifications such as acetylation, which facilitates the recruitment of motor proteins to MTs. Decreased α-tubulin acetylation and increased levels of tubulin-specific histone deacetylase 6 were observed in certain NDD-linked models. Treatment with HDAC6 inhibitor Tubastatin A is thought to counteract MT defects and restore MT-based cargo trafficking.

MTs are composed of α- and β-tubulin heterodimers, which undergo a dynamic process of polymerization (growing state) and depolymerization (shrinking state). After neurite extension during early neurodevelopment, MT stabilization is essential for axon specification and the polarity of developing neurons (Conde and Cáceres, 2009; Hoogenraad and Bradke, 2009). MTs control fundamental processes occurring during neurodevelopment, including intracellular transport, axon guidance, and synapse formation. Stabilized MTs can recruit kinesin-1 motors to initiate polarized trafficking of various organelles and cargos that are necessary for the formation of axons and synapses. Mutations in tubulin genes disturb MT stability leading to abnormalities in brain development commonly referred to as tubulinopathies, including lissencephaly (“smooth brain”), polymicrogyria (“excessive cerebral cortex folding and malformations of cortical layering”), and malformations of cortical development. Strikingly, ∼300 mutations have been described in the genes encoding α- and β-tubulins, including *TUBA1A*, *TUBA1C*, *TUBA4A*, *TUBB1*, *TUBB2A*, *TUBB2B*, *TUBB3*, *TUBB4A*, *TUBB4B*, and *TUBB8*, from patients displaying severe brain malformations associated with ID and refractory childhood epilepsy (Fourel and Boscheron, 2020; Pham and Morrissette, 2019). These mutations are thought to disrupt MT dynamics by switching from growth to shrinkage (depolymerization), which disrupts axon growth, presynaptic cargo transport, and synaptogenesis (Fig. 3). Mutations in tubulin genes, such as *TUBB3*, not only affect MT stability but also reduce kinesin localization to MTs, thus disrupting axonal transport (Minoura et al., 2016; Niwa et al., 2013). Future studies using neurons differentiated from patient-derived induced pluripotent stem cells (iPSCs) will provide direct evidence of how these tubulin mutations affect presynaptic assembly.

### MT modification

MAPs, +TIPs, and signaling proteins involved in posttranslational modifications play critical roles in MT nucleation, assembly, or stability. Mutations in these proteins were reported in patients with lissencephaly, ID, and ASD, resulting in MT destabilization and impaired axonal transport (Fig. 3). For example, *LIS1* is one of the first identified MT-related genes in type-I lissencephaly patients (Reiner et al., 1993). LIS1 increases the MT-binding of dynein and activates dynein by relieving its autoinhibited form, thus facilitating retrograde transport (Htet et al., 2020; Karasmanis et al., 2023). *Lis1*-null mice die prenatally and *Lis1*^*+/−*^ mice display deficits in motor coordination and cognition, as well as severe brain abnormalities (Hirotsune et al., 1998; Youn et al., 2009), while increased LIS1 expression causes severe brain malformation (Bi et al., 2009). CAP-Gly domain-containing linker protein 1 (CLIP1) is localized at +TIP of growing MTs and regulates MT-based axonal transport in developing neurons by recruiting dynein to MTs through its interaction with LIS1 (Coquelle et al., 2002). A mutation deleting *CLIP1* was identified in families of ID patients (Larti et al., 2015).

### Kinesin motors

**Figure 4.**
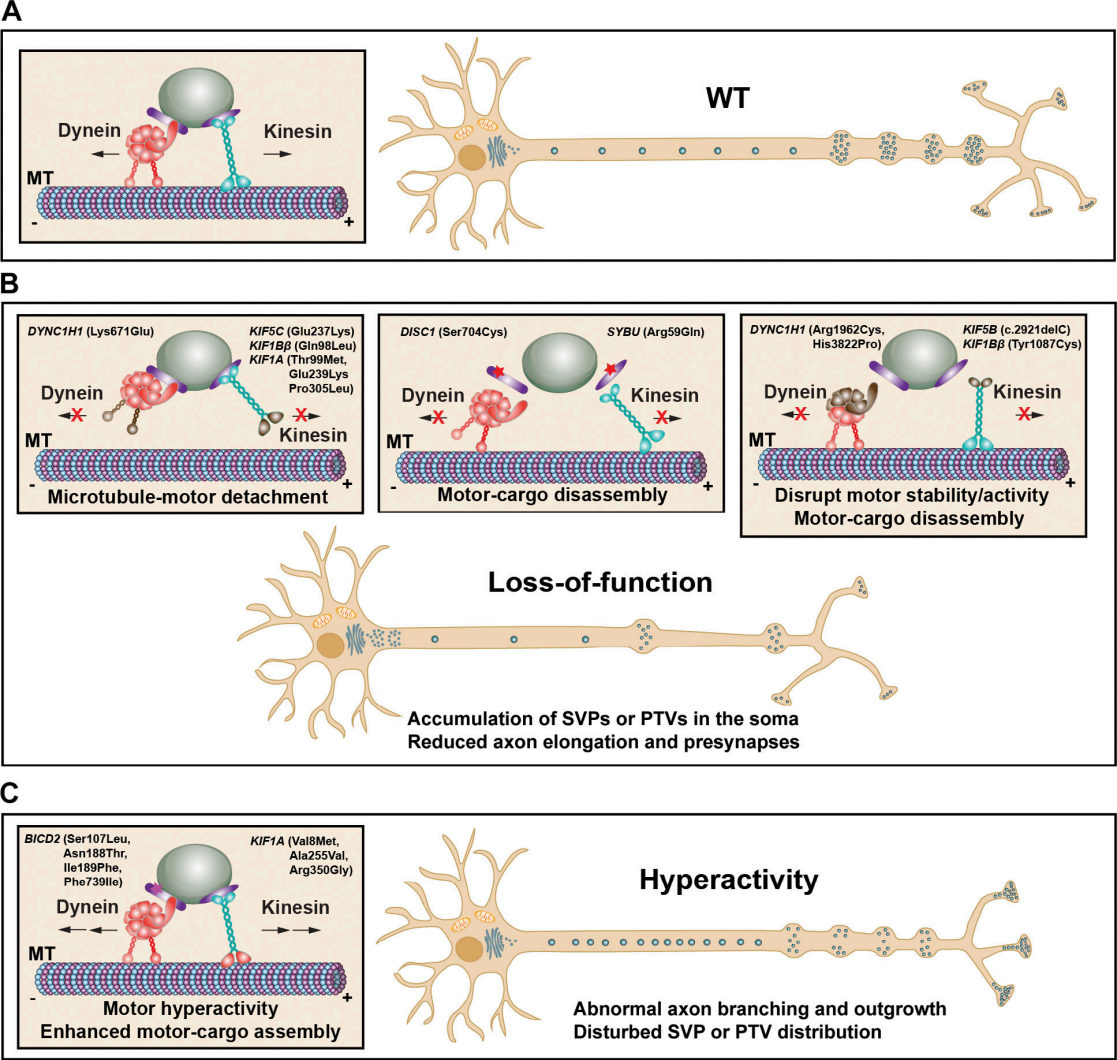
**NDD-linked mutations in motors and adaptors. (A)** Kinesin and dynein are MT-based and ATP-driven molecular motors that move cargos in the anterograde and retrograde directions, respectively. Proper axonal transport is essential for presynaptic assembly. **(B)** Loss-of-function mutations: growing numbers of NDD-linked variants in genes encoding motors and adaptors are loss-of-function mutations that lead to MT-motor detachment, motor-cargo disassembly, or disruption of motor stability and activity, therefore impairing axonal transport of presynaptic cargos, inducing the accumulation of presynaptic components in the somadendritic regions and reduction in axon elongation and presynaptic formation. **(C)** Hyperactive mutations: certain NDD-linked variants in dynein regulatory proteins BICD2 display hyperactive motor activity by enhancing formation of the dynein–dynactin motor complex. Some *KIF1A* variants disrupt its autoinhibition leading to abnormally hyperactive motors and over-transport of SVPs to axon tips, disturbing axon branching and outgrowth as well as the targeted distribution of presynaptic cargos along axons.

The kinesin-3 motor KIF1A mediates axonal transport of a large population of presynaptic cargos, including SVPs, matured SVs, DCVs, as well as some PTVs (Fig. 4 A). Whole-genome sequencing identified more than 100-point mutations in *KIF1A* with dominant and recessive inheritance from patients suffering from a broad spectrum of KIF1A-associated neurological disorder (KAND) (see https://Kif1a.org) (Boyle et al., 2021). *KIF1A* autosomal dominant mutations cause moderate to severe developmental delay with ID and cerebellar atrophy, while recessive mutations lead to progressive spastic paraplegia and hereditary sensory neuropathy. Most variants in *KIF1A* locate in the motor domain with loss-of-function mutations and a few gain-of-function mutations (Chiba et al., 2023; Nair et al., 2023;). Some *KIF1A* variants (Thr99Met, Glu239Lys, and Pro305Leu) disrupt motor activity in a dominant-negative manner, impeding axonal transport and accumulating SVs in the somadendrites with reduction in presynapses (Fig. 4 B) (Anazawa et al., 2022; Morikawa et al., 2022). Other *KIF1A* mutations (Ala255Val, Val8Met, and Arg350Gly) disrupt its autoinhibition, leading to abnormally hyperactive motors and overtransport of SVPs to axon tips but disturb the proper distribution of SVPs to *en passant* synapses in *C. elegans* (Fig. 4 C) (Chiba et al., 2019). Patients carrying Arg13His and Asn222His *KIF1A* mutations are at a high risk of ASD, characterized by moderate to severe deficits in social interaction and communication (Huang et al., 2021; Kurihara et al., 2020). Two heterozygous mutations in *KIF1Bβ* (Gln98Leu and Tyr1087Cys) were identified in patients with Charcot-Marie-Tooth disease type 2A1 (CMT2A1) presenting with childhood-onset motor retardation (Zhao et al., 2001). The Gln98Leu variant resides in the conserved ATP-binding site and significantly reduces ATPase activity, resulting in perinuclear accumulation of mutant *KIF1Bβ.* The Tyr1087Cys variant decreases *KIF1Bβ* cargo binding capacity and impairs the axonal transport of insulin-like growth factor 1 receptor, which is critical for neuronal survival and axonal development (Xu et al., 2018).

For the kinesin-1 family, numerous missense *KIF5A* mutations located in the motor and stalk domains have been linked to spastic paraplegia type 10 (SPG10), a genetic neurodevelopmental disorder (Carosi et al., 2015; Crimella et al., 2012; Méreaux et al., 2022). These KIF5A mutants show abnormal motor ATPase activity and perturbed axonal transport. Three *KIF5A* *de novo* stop-loss frameshift variants were reported in patients suffering from severe infantile-onset myoclonic seizures and early developmental arrest (Duis et al., 2016; Rydzanicz et al., 2017). These mutations either cause truncation (921delC) or abnormal elongation (2854delC and 2934delG) of the *KIF5A* C-terminus. A whole exome sequencing study revealed a homozygous *KIF5B* variant (His751Arg) in a family with brain malformation and ID (Charng et al., 2016). A heterozygous variant in the *KIF5C* motor domain (Glu237Lys) was reported in patients with severe ID (de Ligt et al., 2012; Poirier et al., 2013).

The kinesin-2 motor KIF3 is one of the most abundantly expressed KIFs in the nervous system (Kondo et al., 1994). KIF3 is a heterotrimer containing two distinct heavy chains, KIF3A and KIF3B, and one light chain, KAP3. KIF3 plays a role in neurite elongation and branching by transporting a variety of organelles, including fodrin-associated vesicles and collapsin response mediator protein 2 (CRMP2)-containing vesicles (Takeda et al., 2000; Yoshihara et al., 2021). A *KIF3B* nonsense mutation (Arg654Ter) was found in SZ patients (Alsabban et al., 2020). Neurite hyperbranching resulting from impaired transport of CRMP2-containing vesicles is a causative mechanism of KIF3B-related SZ pathogenesis. KIF3 expression is reduced in brains of patients with SZ. *Kif3b*^*+/−*^ mutant mice display a range of behavioral characteristics of SZ, and KIF3B (Arg654Ter) mutant protein fails to rescue the cellular phenotypes observed in *Kif3b*^*+/−*^ neurons (Yoshihara et al., 2021).

### Dynein motors

Over 100 *de novo* heterozygous mutations in the *DYNC1H1* gene have been identified in patients with malformations of cortical development and/or developmental delay and ID (Ge et al., 2023; Poirier et al., 2013; Willemsen et al., 2012), as well as early childhood-onset motor retardation (Jamuar et al., 2014; Weedon et al., 2011), or a combination of these phenotypes. These variants are scattered throughout the dynein motor stem, motor, and neck domains and have a dominant-negative or gain-of-function effect (Amabile et al., 2020; Hoang et al., 2017). Functional analysis of 14 *DYNC1H1* patient mutations showed that most mutations result in impaired motility due to reduced dynein expression/stability (Lys671Glu) or compromised processivity (Arg1962Cys and His3822Pro) (Fig. 4 B). A combination of clinical, molecular, and cellular investigations will provide mechanistic insights into the relationships between axonal transport defects in *DYNC1H1* mutations and presynaptic assembly/maturation in NDDs.

### Motor adaptors and effectors

Kinesin-binding protein (KBP, KIF1BP) directly binds to the motor domain of KIF1A and KIF1B. This binding inhibits motor-MT attachment and thus interferes with cargo transport (Atherton et al., 2020). While overexpressing KBP inhibits axonal transport of SVPs in cultured hippocampal neurons and in *C. elegans* sensory neurons (Kevenaar et al., 2016), depleting KBP results in abnormal KIF1A and SV accumulation in axonal growth cones. Mice lacking KBP die shortly after birth with smaller brains. In addition, KBP can alter MT dynamics by inhibiting MT-depolymerizing kinesins, such as KIF18A. Human genetic data shows that homozygous mutations in *KBP* are linked to Goldberg-Shprintzen syndrome (GOSHS) distinguished by ID, microcephaly, and axonal neuropathy (Brooks et al., 2005; Valence et al., 2013; Salehpour et al., 2017).

Our previous studies provided direct evidence supporting an axonal transport mechanism underlying autism-like synaptic dysfunction and social behavioral traits. Syntabulin functions as a kinesin-1 adaptor driving PTV transport for synapse assembly and maintenance (Cai et al., 2007; Su et al., 2004). Syntabulin expression in mouse brains peaks during the first 2 wk after birth and then progressively declines with brain maturation (Xiong et al., 2021). The syntabulin gene (*SYBU*) is located within the autism susceptibility loci 8q22-24 (Chen et al., 2017; Sánchez Delgado et al., 2014). A recent whole exome sequencing study identified an autism-linked *de novo* missense variant (Arg59Gln) in the human *SYBU* gene (Herman et al., 2016). These findings raise a fundamental question of a mechanistic link between defects in syntabulin-mediated PTV transport and autism-like phenotypes. We recently demonstrated striking phenotypes in *Sybu* conditional knockout (cKO) mice: (1) impaired transport of PTVs from the soma towards distal axons, (2) reduced density of presynapses and AZs in mouse brains, and (3) altered synaptic transmission and plasticity (Fig. 4 B). Intriguingly, *Sybu* cKO mice also exhibit core autism-like traits, including defective social recognition and communication, increased stereotypic behavior, and impaired spatial learning and memory (Xiong et al., 2021). Functional studies confirmed that the autism-linked missense variant (Arg59Gln) loses its adapter capacity for binding kinesin-1 motors and thus impairs anterograde axonal transport of PTVs. These phenotypes establish for the first time that human autism-linked deficits in axonal PTV transport contribute to autism-like behavioral abnormalities.

Disease-linked mutations in dynein regulatory proteins, including BICD2 and nuclear distribution protein E1 (NDE1), are also associated with a broad phenotypic spectrum from early-onset peripheral neuropathies to malformations of cortical development (Lipka et al., 2013). BICD2 binds to the dynein–dynactin motor complex through its coiled-coil domains and thus modulates dynein-driven cargo transport, such as mRNA and Rab6-positive secretory vesicles (Grigoriev et al., 2007; Sladewski et al., 2018). Dominant missense mutations in *BICD2* were linked to prenatal/childhood-onset spinal muscular atrophy (SMALED), a developmental disease of motor neurons affecting the lower limbs (Neveling et al., 2013; Rossor et al., 2015; Storbeck et al., 2017; Trimouille et al., 2018). Certain *BICD2* mutants (Ser107Leu, Asn188Thr, Ile189Phe, and Phe739Ile) display hyperactivated motors by enhancing dynein–dynactin motor complex formation (Fig. 4 C). However, increased retrograde transport blocks neurite outgrowth in rat hippocampal neurons (Huynh and Vale, 2017). These studies suggest that an imbalanced anterograde versus retrograde axonal transport may underlie NDDs.

The fragile X mental retardation protein (FMRP) is an mRNA-binding protein involved in the transport, localization, and translational regulation of a subset of dendritic mRNAs (Grossman et al., 2006); its loss is associated with ID and ASD (O’Donnell and Warren, 2002). FMRP and its homologs, FXR1P and FXR2P, are highly expressed in the developing brain and observed in the form of discrete granules (fragile X granules) localized in axons and presynaptic terminals in a set of mouse brain regions (Christie et al., 2009). FMRP loss leads to cell-autonomous defects in presynaptic terminal formation in organotypic mouse hippocampal slices (Hanson and Madison, 2007). Interestingly, BICD2 colocalizes with FMRP transport particles and facilitates their bidirectional trafficking (Bianco et al., 2010), which may also involve kinesin-1 and dynein motors (Splinter et al., 2010). FMRP protein levels are reduced in neurons overexpressing disease variant *BICD2* (Lys730Met), which displays reduced FMRP puncta trafficking into processes leading to abnormal neuron morphogenesis (Bianco et al., 2010).

NDE1 and its ortholog NDEL1 work together with LIS1 to promote dynein motor activity (Garrott et al., 2022). Truncating mutations in *NDE1* were reported in patients with lissencephaly-4 (Alkuraya et al., 2011; Bakircioglu et al., 2011). As a DISC1 binding partner, NED1/NDEL1 may also play a role in the etiology of SZ (Burdick et al., 2008). Rare *NED1* mutations within exon 7 contribute to SZ susceptibility by affecting axonal outgrowth (Kimura et al., 2015). NDEL1, FEZ1, and LIS1 expression levels are significantly reduced in the hippocampus of SZ patients carrying *DISC1* polymorphisms (Ser704Cys) (Lipska et al., 2006). These genetic studies support the emerging concept that disruptions in presynaptic cargo transport are the earliest contributors to the pathogenesis of a broad spectrum of NDDs; therefore, restoration of these transport defects is an attractive therapeutic strategy.

## Therapeutic restoration of defective axonal transport in NDDs

At present, significant attention is focused on three promising themes: (1) stabilizing MTs to restore trafficking tracks; (2) targeting signaling pathways to modify transport machinery; and (3) gene-based editing to correct mutations in tubulin, motors, and adaptors.

### MT stabilizers

A well-stabilized MT network constitutes a base for efficient axonal transport. Alterations in MT dynamics are one of the major causative mechanisms for many NDDs. MT-stabilizing agents have shown some beneficial effects on ameliorating neurological and behavioral deficits in various models of NDDs, including ID, ASD, SZ, and epilepsy (Fig. 3) (Bonini et al., 2017; Gambino et al., 2022; Liaci et al., 2021). For example, Epothilone D (EpoD), a taxol-related compound that interacts with tubulin to stabilize MTs, ameliorates synaptic function and behavior in mouse models of neuropathy and SZ (Andrieux et al., 2006; Brunden et al., 2010). At nanomolar concentrations, EpoD improves MT density and axonal transport, reduces axonal dystrophy, and enhances cognitive performance (Zhang et al., 2012). MT stabilization can also be achieved by targeting MT-regulating proteins. Calpain inhibitors that protect LIS1 from proteolysis can recover retrograde transport and improve behavioral performance in *LIS1*^*+/−*^ mice (Toba et al., 2013; Yamada et al., 2009). Alterations in tubulin posttranslational modifications have been reported in certain forms of NDDs (Moutin et al., 2021). For example, Rett Syndrome (RTT), a severe NDD with ID, autistic features, and motor dysfunction, is caused by loss-of-function mutations in the X-linked methyl-CpG-binding protein 2 (MECP2). Decreased acetylated α-tubulin levels, but increased tubulin-specific histone deacetylase 6 (HDAC6) levels, were observed in *Mecp2*-deficient cells (Gold et al., 2015). Treatment with the HDAC6 inhibitor Tubastatin A can counteract MT defects and thus restore the recruitment of kinesin-1 and dynein motors to promote MT-based cargo trafficking. As promising results are emerging in animal models and clinical trials, MT-targeted interventions represent an attractive therapeutic opportunity.

### Targeting signaling pathways

NDD-linked mutations in genes encoding motors and adaptors also contribute to altered axonal transport. Therefore, the signaling modulation of these motors or adaptors is another therapeutic approach to restore axonal transport. Several protein kinases that directly phosphorylate motors and adaptors are suggested as potential targets. For example, GSK3β, involved in regulation of both kinesin-1 and dynein-driven axonal transport (Du et al., 2010; Gao et al., 2015), was reported to be upregulated in a subset of NDDs. Increased GSK3β activity appeared to disrupt axonal transport through the dissociation of motors and their cargos via phosphorylating KLC and DIC, respectively (Banerjee et al., 2021; Dolma et al., 2014). In addition, treatment with lithium or GSK3β inhibitors suppress GSK3β activity and thus corrects behavioral phenotypes in animal models of NDDs, including ID, ASD, SZ, and epilepsy (Fuchs et al., 2015; Guo et al., 2012; Mao et al., 2009). In RTT, BDNF signaling is disrupted through an HTT-mediated transport mechanism. Surprisingly, promoting HTT-Ser421 phosphorylation by FK506, a calcineurin inhibitor, restores axonal transport of BDNF-carrying SEs and thus improves behavioral phenotypes and survival of *Mecp2* knockout mice (Ehinger et al., 2020). These studies suggest that restoring axonal transport by targeting signaling pathways is an alternative approach, although off-target effects and crosstalk of signaling cascades need to be considered.

### Gene therapy

Thousands of genetic mutations have been associated with distinct types of NDDs. Given that the exact mechanism, timing, and progression of the molecular pathology are largely unknown, 90% of rare NDDs do not currently have an approved treatment. Gene-based editing opens a new avenue for the treatment of NDDs through expression of exogenous or suppression of endogenous genes. Gene therapy has been recently shown to be effective in various animal models of NDDs, such as ASD and epilepsy (Megagiannis et al., 2022; Ozlu et al., 2021; Turner et al., 2021), with several strategies moving toward clinical trials. We previously showed that axonal retrograde transport could be rescued via AAV9-based gene delivery of dynein adaptor Snapin, which reduces disease progression in Amyotrophic Lateral Sclerosis (ALS) mice model (Xie et al., 2015). Recent technological innovations, including improved therapeutic delivery of genetic material and the development of *in vivo* CRISPR-based gene editing, will further improve the feasibility of personalized gene therapy that corrects causative gene mutations to reverse defective axonal transport in NDDs.

## Conclusions and perspectives

NDDs are recognized as one class of “synaptic disorders” where alterations in synaptic structures and functions impair both local and global brain connectivity and information processing. Proper synaptic transmission requires seamless integration of biogenesis, sorting, transport, and assembly of presynaptic components, and maintenance and remodeling of synaptic structures. While synapse development is regulated in multiple steps, the targeted delivery of presynaptic cargos is vital to building and maintaining functional synapses, which requires intricate mechanisms to orchestrate bidirectional transport of various cargos between cell bodies and axon terminals. Recent studies have started to uncover presynaptic mechanisms underlying NDD-linked axonal transport defects. Mutations in genes encoding the axonal transport machinery disturb axonal trafficking in early developmental stages contributing to “synaptopathies,” one of the predominant mechanisms underlying NDDs. A particular area of future investigations will be the molecular compositions and identity of various presynaptic cargos: specifically, (1) how a unique presynaptic cargo can be sorted and packaged in the soma and loaded by a specific set of transport motors and adaptors, (2) how motors initiate and terminate their transport, (3) how motor activity is controlled by local cues within the axon and how motors unload cargos with high precision at thousands of *en passant* boutons and terminal presynapses, (4) whether multiple CAZ and SV proteins package into one transport cargo or cluster together for cotransport, and (5) whether all CAZ components arrive at nascent synapses simultaneously or sequentially. Addressing these fundamental questions will continue to build a more coherent view of mechanisms that maintain synaptic assembly, maturation, and remodeling during brain development and throughout life. Although genetic screenings have identified a growing list of NDD-linked mutations in genes encoding the transport machinery, pathological mechanisms related to these disease variants are rarely studied. As we learn more about the specific interactions among MTs, motors, adaptors, and cargos being transported, the pathobiology induced by these NDD-linked mutations may become clearer. Future studies using advanced single-molecule live imaging and *in vivo* approaches, combined with high-throughput genomics and proteomics, will provide new insights into NDD-linked causative mechanisms underlying axon transport defects of presynaptic cargos during neurodevelopment. Knowledge from human iPSC-derived models, along with gene-editing approaches, could have significant impact on the development of potential therapeutic restoration of defective axonal transport for currently incurable NDDs.

## References

[bib1] Abdel-Hamid, M.S., S.H. El-Dessouky, M.I. Ateya, H.M. Gaafar, and G.M.H. Abdel-Salam. 2019. Phenotypic spectrum of NDE1-related disorders: From microlissencephaly to microhydranencephaly. Am. J. Med. Genet. A. 179:494–497. 10.1002/ajmg.a.61035 30637988

[bib2] Ackermann, F., C.L. Waites, and C.C. Garner. 2015. Presynaptic active zones in invertebrates and vertebrates. EMBO Rep. 16:923–938. 10.15252/embr.201540434 26160654 PMC4552486

[bib3] Ahmari, S.E., J. Buchanan, and S.J. Smith. 2000. Assembly of presynaptic active zones from cytoplasmic transport packets. Nat. Neurosci. 3:445–451. 10.1038/74814 10769383

[bib4] Ailion, M., M. Hannemann, S. Dalton, A. Pappas, S. Watanabe, J. Hegermann, Q. Liu, H.F. Han, M. Gu, M.Q. Goulding, . 2014. Two Rab2 interactors regulate dense-core vesicle maturation. Neuron. 82:167–180. 10.1016/j.neuron.2014.02.017 24698274 PMC3997996

[bib5] Akhmanova, A., and M.O. Steinmetz. 2008. Tracking the ends: A dynamic protein network controls the fate of microtubule tips. Nat. Rev. Mol. Cell Biol. 9:309–322. 10.1038/nrm2369 18322465

[bib6] Akins, M.R., H.E. Berk-Rauch, and J.R. Fallon. 2009. Presynaptic translation: Stepping out of the postsynaptic shadow. Front. Neural. Circuits. 4:3–17. 10.3389/neuro.04.017.2009 PMC2776480 19915727

[bib7] Alkuraya, F.S., X. Cai, C. Emery, G.H. Mochida, M.S. Al-Dosari, J.M. Felie, R.S. Hill, B.J. Barry, J.N. Partlow, G.G. Gascon, . 2011. Human mutations in NDE1 cause extreme microcephaly with lissencephaly [corrected]. Am. J. Hum. Genet. 88:536–547. 10.1016/j.ajhg.2011.04.003 21529751 PMC3146728

[bib8] Alsabban, A.H., M. Morikawa, Y. Tanaka, Y. Takei, and N. Hirokawa. 2020. Kinesin Kif3b mutation reduces NMDAR subunit NR2A trafficking and causes schizophrenia-like phenotypes in mice. EMBO J. 39:e101090. 10.15252/embj.2018101090 31746486 PMC6939202

[bib9] Altar, C.A., N. Cai, T. Bliven, M. Juhasz, J.M. Conner, A.L. Acheson, R.M. Lindsay, and S.J. Wiegand. 1997. Anterograde transport of brain-derived neurotrophic factor and its role in the brain. Nature. 389:856–860. 10.1038/39885 9349818

[bib10] Amabile, S., L. Jeffries, J.M. McGrath, W. Ji, M. Spencer-Manzon, H. Zhang, and S.A. Lakhani. 2020. DYNC1H1-related disorders: A description of four new unrelated patients and a comprehensive review of previously reported variants. Am. J. Med. Genet. A. 182:2049–2057. 10.1002/ajmg.a.61729 32656949

[bib11] Anazawa, Y., T. Kita, R. Iguchi, K. Hayashi, and S. Niwa. 2022. De novo mutations in KIF1A-associated neuronal disorder (KAND) dominant-negatively inhibit motor activity and axonal transport of synaptic vesicle precursors. Proc. Natl. Acad. Sci. USA. 119:e2113795119. 10.1073/pnas.2113795119 35917346 PMC9371658

[bib12] Andreassi, C., and A. Riccio. 2009. To localize or not to localize: mRNA fate is in 3′UTR ends. Trends Cell Biol. 19:465–474. 10.1016/j.tcb.2009.06.001 19716303

[bib290] Andres-Alonso, M, M.R. Ammar, I. Butnaru, G.M. Gomes, . 2019. SIPA1L2 controls trafficking and local signaling of TrkB-containing amphisomes at presynaptic terminals. Nat. Commun. 10:5448. 10.1038/s41467-019-13224-z 31784514 PMC6884526

[bib13] Andrieux, A., P. Salin, A. Schweitzer, M. Bégou, B. Pachoud, P. Brun, S. Gory-Fauré, P. Kujala, M.F. Suaud-Chagny, G. Höfle, and D. Job. 2006. Microtubule stabilizer ameliorates synaptic function and behavior in a mouse model for schizophrenia. Biol. Psychiatry. 60:1224–1230. 10.1016/j.biopsych.2006.03.048 16806091

[bib14] Arimura, N., T. Kimura, S. Nakamuta, S. Taya, Y. Funahashi, A. Hattori, A. Shimada, C. Ménager, S. Kawabata, K. Fujii, . 2009. Anterograde transport of TrkB in axons is mediated by direct interaction with Slp1 and Rab27. Dev. Cell. 16:675–686. 10.1016/j.devcel.2009.03.005 19460344

[bib15] Ascaño, M., A. Richmond, P. Borden, and R. Kuruvilla. 2009. Axonal targeting of Trk receptors via transcytosis regulates sensitivity to neurotrophin responses. J. Neurosci. 29:11674–11685. 10.1523/JNEUROSCI.1542-09.2009 19759314 PMC2775807

[bib16] Atherton, J., J.J. Hummel, N. Olieric, J. Locke, A. Peña, S.S. Rosenfeld, M.O. Steinmetz, C.C. Hoogenraad, and C.A. Moores. 2020. The mechanism of kinesin inhibition by kinesin-binding protein. Elife. 9:e61481. 10.7554/eLife.61481 33252036 PMC7746232

[bib17] Badal, K.K., and S.V. Puthanveettil. 2022. Axonal transport deficits in neuropsychiatric disorders. Mol. Cell. Neurosci. 123:103786. 10.1016/j.mcn.2022.103786 36252719 PMC12717855

[bib296] Bakircioglu, M., O.P. Carvalho, M. Khurshid, J.J. Cox, B. Tuysuz, . 2011. The essential role of centrosomal NDE1 in human cerebral cortex neurogenesis. Am. J. Hum. Genet. 88:523–535. 10.1016/j.ajhg.2011.03.019 21529752 PMC3146716

[bib18] Banerjee, R., P. Chakraborty, M.C. Yu, and S. Gunawardena. 2021. A stop or go switch: Glycogen synthase kinase 3β phosphorylation of the kinesin 1 motor domain at Ser314 halts motility without detaching from microtubules. Development. 148:dev199866. 10.1242/dev.199866 34940839 PMC8722386

[bib19] Batista, A.F.R., J.C. Martínez, and U. Hengst. 2017. Intra-axonal synthesis of SNAP25 is required for the formation of presynaptic terminals. Cell Rep. 20:3085–3098. 10.1016/j.celrep.2017.08.097 28954226 PMC5659736

[bib20] Bentley, M., H. Decker, J. Luisi, and G. Banker. 2015. A novel assay reveals preferential binding between Rabs, kinesins, and specific endosomal subpopulations. J. Cell Biol. 208:273–281. 10.1083/jcb.201408056 25624392 PMC4315250

[bib21] Bharat, V., M. Siebrecht, K. Burk, S. Ahmed, C. Reissner, M. Kohansal-Nodehi, V. Steubler, M. Zweckstetter, J.T. Ting, and C. Dean. 2017. Capture of dense core vesicles at synapses by JNK-dependent phosphorylation of synaptotagmin-4. Cell Rep. 21:2118–2133. 10.1016/j.celrep.2017.10.084 29166604 PMC5714612

[bib22] Bi, W., T. Sapir, O.A. Shchelochkov, F. Zhang, M.A. Withers, J.V. Hunter, T. Levy, V. Shinder, D.A. Peiffer, K.L. Gunderson, . 2009. Increased LIS1 expression affects human and mouse brain development. Nat. Genet. 41:168–177. 10.1038/ng.302 19136950 PMC4396744

[bib23] Bianco, A., M. Dienstbier, H.K. Salter, G. Gatto, and S.L. Bullock. 2010. Bicaudal-D regulates fragile X mental retardation protein levels, motility, and function during neuronal morphogenesis. Curr. Biol. 20:1487–1492. 10.1016/j.cub.2010.07.016 20691595 PMC2927779

[bib24] Bittins, C.M., T.W. Eichler, J.A. Hammer III, and H.H. Gerdes. 2010. Dominant-negative myosin Va impairs retrograde but not anterograde axonal transport of large dense core vesicles. Cell. Mol. Neurobiol. 30:369–379. 10.1007/s10571-009-9459-2 19787448 PMC3878150

[bib25] Blasius, T.L., D. Cai, G.T. Jih, C.P. Toret, and K.J. Verhey. 2007. Two binding partners cooperate to activate the molecular motor Kinesin-1. J. Cell Biol. 176:11–17. 10.1083/jcb.200605099 17200414 PMC2063617

[bib26] Blondeau, F., B. Ritter, P.D. Allaire, S. Wasiak, M. Girard, N.K. Hussain, A. Angers, V. Legendre-Guillemin, L. Roy, D. Boismenu, . 2004. Tandem MS analysis of brain clathrin-coated vesicles reveals their critical involvement in synaptic vesicle recycling. Proc. Natl. Acad. Sci. USA. 101:3833–3838. 10.1073/pnas.0308186101 15007177 PMC374330

[bib27] Bonini, S.A., A. Mastinu, G. Ferrari-Toninelli, and M. Memo. 2017. Potential role of microtubule stabilizing agents in neurodevelopmental disorders. Int. J. Mol. Sci. 18:1627. 10.3390/ijms18081627 28933765 PMC5578018

[bib28] Bourgeron, T. 2015. From the genetic architecture to synaptic plasticity in autism spectrum disorder. Nat. Rev. Neurosci. 16:551–563. 10.1038/nrn3992 26289574

[bib29] Boyle, L., L. Rao, S. Kaur, X. Fan, C. Mebane, L. Hamm, A. Thornton, J.T. Ahrendsen, M.P. Anderson, J. Christodoulou, . 2021. Genotype and defects in microtubule-based motility correlate with clinical severity in KIF1A-associated neurological disorder. HGG Adv. 2:100026. 10.1016/j.xhgg.2021.100026 33880452 PMC8054982

[bib30] Breuss, M., J.I. Heng, K. Poirier, G. Tian, X.H. Jaglin, Z. Qu, A. Braun, T. Gstrein, L. Ngo, M. Haas, . 2012. Mutations in the β-tubulin gene TUBB5 cause microcephaly with structural brain abnormalities. Cell Rep. 2:1554–1562. 10.1016/j.celrep.2012.11.017 23246003 PMC3595605

[bib31] Brooks, A.S., A.M. Bertoli-Avella, G.M. Burzynski, G.J. Breedveld, J. Osinga, L.G. Boven, J.A. Hurst, G.M. Mancini, M.H. Lequin, R.F. de Coo, . 2005. Homozygous nonsense mutations in KIAA1279 are associated with malformations of the central and enteric nervous systems. Am. J. Hum. Genet. 77:120–126. 10.1086/431244 15883926 PMC1226183

[bib32] Brunden, K.R., B. Zhang, J. Carroll, Y. Yao, J.S. Potuzak, A.M. Hogan, M. Iba, M.J. James, S.X. Xie, C. Ballatore, . 2010. Epothilone D improves microtubule density, axonal integrity, and cognition in a transgenic mouse model of tauopathy. J. Neurosci. 30:13861–13866. 10.1523/JNEUROSCI.3059-10.2010 20943926 PMC2958430

[bib33] Buckmaster, P.S., H.J. Wenzel, D.D. Kunkel, and P.A. Schwartzkroin. 1996. Axon arbors and synaptic connections of hippocampal mossy cells in the rat in vivo. J. Comp. Neurol. 366:271–292. 10.1002/(sici)1096-9861(19960304)366:2<270:aid-cne7>3.0.co;2-2 8698887

[bib34] Budzinska, M.I., D. Villarroel-Campos, M. Golding, A. Weston, L. Collinson, A.P. Snijders, and G. Schiavo. 2020. PTPN23 binds the dynein adaptor BICD1 and is required for endocytic sorting of neurotrophin receptors. J. Cell Sci. 133:jcs242412. 10.1242/jcs.242412 32079660 PMC7132798

[bib35] Burdick, K.E., A. Kamiya, C.A. Hodgkinson, T. Lencz, P. DeRosse, K. Ishizuka, S. Elashvili, H. Arai, D. Goldman, A. Sawa, and A.K. Malhotra. 2008. Elucidating the relationship between DISC1, NDEL1 and NDE1 and the risk for schizophrenia: Evidence of epistasis and competitive binding. Hum. Mol. Genet. 17:2462–2473. 10.1093/hmg/ddn146 18469341 PMC2486442

[bib36] Bury, L.A.D., and S.L. Sabo. 2011. Coordinated trafficking of synaptic vesicle and active zone proteins prior to synapse formation. Neural Dev. 6:24. 10.1186/1749-8104-6-24 21569270 PMC3103415

[bib37] Cai, Q., P.Y. Pan, and Z.H. Sheng. 2007. Syntabulin-kinesin-1 family member 5B-mediated axonal transport contributes to activity-dependent presynaptic assembly. J. Neurosci. 27:7284–7296. 10.1523/JNEUROSCI.0731-07.2007 17611281 PMC6794594

[bib38] Cajigas, I.J., G. Tushev, T.J. Will, S. tom Dieck, N. Fuerst, and E.M. Schuman. 2012. The local transcriptome in the synaptic neuropil revealed by deep sequencing and high-resolution imaging. Neuron. 74:453–466. 10.1016/j.neuron.2012.02.036 22578497 PMC3627340

[bib39] Carosi, L., T. Lo Giudice, M. Di Lullo, F. Lombardi, C. Babalini, F. Gaudiello, G.A. Marfia, R. Massa, T. Kawarai, and A. Orlacchio. 2015. Hereditary spastic paraplegia: A novel mutation and expansion of the phenotype variability in SPG10. J. Neurol. Neurosurg. Psychiatry. 86:702–704. 10.1136/jnnp-2014-308625 25352184 PMC4453490

[bib40] Cason, S.E., and E.L.F. Holzbaur. 2022. Selective motor activation in organelle transport along axons. Nat. Rev. Mol. Cell Biol. 23:699–714. 10.1038/s41580-022-00491-w 35637414 PMC12998404

[bib41] Caviston, J.P., J.L. Ross, S.M. Antony, M. Tokito, and E.L.F. Holzbaur. 2007. Huntingtin facilitates dynein/dynactin-mediated vesicle transport. Proc. Natl. Acad. Sci. USA. 104:10045–10050. 10.1073/pnas.0610628104 17548833 PMC1891230

[bib42] Cavolo, S.L., D. Bulgari, D.L. Deitcher, and E.S. Levitan. 2016. Activity induces Fmr1-sensitive synaptic capture of anterograde circulating neuropeptide vesicles. J. Neurosci. 36:11781–11787. 10.1523/JNEUROSCI.2212-16.2016 27852784 PMC5125230

[bib43] Cederquist, G.Y., A. Luchniak, M.A. Tischfield, M. Peeva, Y. Song, M.P. Menezes, W.M. Chan, C. Andrews, S. Chew, R.V. Jamieson, . 2012. An inherited TUBB2B mutation alters a kinesin-binding site and causes polymicrogyria, CFEOM and axon dysinnervation. Hum. Mol. Genet. 21:5484–5499. 10.1093/hmg/dds393 23001566 PMC3516133

[bib44] Celestino, R., J.B. Gama, A.F. Castro-Rodrigues, D.J. Barbosa, H. Rocha, E.A. d’Amico, A. Musacchio, A.X. Carvalho, J.H. Morais-Cabral, and R. Gassmann. 2022. JIP3 interacts with dynein and kinesin-1 to regulate bidirectional organelle transport. J. Cell Biol. 221:e202110057. 10.1083/jcb.202110057 35829703 PMC9284427

[bib45] Chao, M.V. 2003. Neurotrophins and their receptors: A convergence point for many signalling pathways. Nat. Rev. Neurosci. 4:299–309. 10.1038/nrn1078 12671646

[bib46] Charng, W.L., E. Karaca, Z. Coban Akdemir, T. Gambin, M.M. Atik, S. Gu, J.E. Posey, S.N. Jhangiani, D.M. Muzny, H. Doddapaneni, . 2016. Exome sequencing in mostly consanguineous Arab families with neurologic disease provides a high potential molecular diagnosis rate. BMC Med. Genomics. 9:42. 10.1186/s12920-016-0208-3 27435318 PMC4950750

[bib47] Chen, C.H., H.I. Chen, W.H. Chien, L.H. Li, Y.Y. Wu, Y.N. Chiu, W.C. Tsai, and S.S. Gau. 2017. High resolution analysis of rare copy number variants in patients with autism spectrum disorder from Taiwan. Sci. Rep. 7:11919. 10.1038/s41598-017-12081-4 28931914 PMC5607249

[bib48] Cheng, P.L., A.H. Song, Y.H. Wong, S. Wang, X. Zhang, and M.M. Poo. 2011. Self-amplifying autocrine actions of BDNF in axon development. Proc. Natl. Acad. Sci. USA. 108:18430–18435. 10.1073/pnas.1115907108 22025720 PMC3215064

[bib49] Chia, P.H., P. Li, and K. Shen. 2013. Cell biology in neuroscience: Cellular and molecular mechanisms underlying presynapse formation. J. Cell Biol. 203:11–22. 10.1083/jcb.201307020 24127213 PMC3798257

[bib50] Chiba, K., H. Takahashi, M. Chen, H. Obinata, S. Arai, K. Hashimoto, T. Oda, R.J. McKenney, and S. Niwa. 2019. Disease-associated mutations hyperactivate KIF1A motility and anterograde axonal transport of synaptic vesicle precursors. Proc. Natl. Acad. Sci. USA. 116:18429–18434. 10.1073/pnas.1905690116 31455732 PMC6744892

[bib51] Chiba, K., T. Kita, Y. Anazawa, and S. Niwa. 2023. Insight into the regulation of axonal transport from the study of KIF1A-associated neurological disorder. J. Cell Sci. 136:jcs260742. 10.1242/jcs.260742 36655764

[bib52] Christensen, J.R., A.A. Kendrick, J.B. Truong, A. Aguilar-Maldonado, V. Adani, M. Dzieciatkowska, and S.L. Reck-Peterson. 2021. Cytoplasmic dynein-1 cargo diversity is mediated by the combinatorial assembly of FTS-Hook-FHIP complexes. Elife. 10:e74538. 10.7554/eLife.74538 34882091 PMC8730729

[bib53] Christie, S.B., M.R. Akins, J.E. Schwob, and J.R. Fallon. 2009. The FXG: A presynaptic fragile X granule expressed in a subset of developing brain circuits. J. Neurosci. 29:1514–1524. 10.1523/JNEUROSCI.3937-08.2009 19193898 PMC2746438

[bib54] Chua, J.J., E. Butkevich, J.M. Worseck, M. Kittelmann, M. Grønborg, E. Behrmann, U. Stelzl, N.J. Pavlos, M.M. Lalowski, S. Eimer, . 2012. Phosphorylation-regulated axonal dependent transport of syntaxin 1 is mediated by a Kinesin-1 adapter. Proc. Natl. Acad. Sci. USA. 109:5862–5867. 10.1073/pnas.1113819109 22451907 PMC3326461

[bib55] Cingolani, L.A., and Y. Goda. 2008. Actin in action: The interplay between the actin cytoskeleton and synaptic efficacy. Nat. Rev. Neurosci. 9:344–356. 10.1038/nrn2373 18425089

[bib56] Cioni, J.M., J.Q. Lin, A.V. Holtermann, M. Koppers, M.A.H. Jakobs, A. Azizi, B. Turner-Bridger, T. Shigeoka, K. Franze, W.A. Harris, and C.E. Holt. 2019. Late endosomes act as mRNA translation platforms and sustain mitochondria in axons. Cell. 176:56–72.e15. 10.1016/j.cell.2018.11.030 30612743 PMC6333918

[bib57] Citterio, A., A. Arnoldi, E. Panzeri, L. Merlini, M.G. D’Angelo, O. Musumeci, A. Toscano, A. Bondi, A. Martinuzzi, N. Bresolin, and M.T. Bassi. 2015. Variants in KIF1A gene in dominant and sporadic forms of hereditary spastic paraparesis. J. Neurol. 262:2684–2690. 10.1007/s00415-015-7899-9 26410750

[bib58] Cohen, L.D., R. Zuchman, O. Sorokina, A. Müller, D.C. Dieterich, J.D. Armstrong, T. Ziv, and N.E. Ziv. 2013. Metabolic turnover of synaptic proteins: Kinetics, interdependencies and implications for synaptic maintenance. PLoS One. 8:e63191. 10.1371/journal.pone.0063191 23658807 PMC3642143

[bib59] Colin, E., D. Zala, G. Liot, H. Rangone, M. Borrell-Pagès, X.J. Li, F. Saudou, and S. Humbert. 2008. Huntingtin phosphorylation acts as a molecular switch for anterograde/retrograde transport in neurons. EMBO J. 27:2124–2134. 10.1038/emboj.2008.133 18615096 PMC2516882

[bib60] Conde, C., and A. Cáceres. 2009. Microtubule assembly, organization and dynamics in axons and dendrites. Nat. Rev. Neurosci. 10:319–332. 10.1038/nrn2631 19377501

[bib61] Coquelle, F.M., M. Caspi, F.P. Cordelières, J.P. Dompierre, D.L. Dujardin, C. Koifman, P. Martin, C.C. Hoogenraad, A. Akhmanova, N. Galjart, . 2002. LIS1, CLIP-170's key to the dynein/dynactin pathway. Mol. Cell. Biol. 22:3089–3102. 10.1128/MCB.22.9.3089-3102.2002 11940666 PMC133759

[bib62] Crimella, C., C. Baschirotto, A. Arnoldi, A. Tonelli, E. Tenderini, G. Airoldi, A. Martinuzzi, A. Trabacca, L. Losito, M. Scarlato, . 2012. Mutations in the motor and stalk domains of KIF5A in spastic paraplegia type 10 and in axonal Charcot-Marie-Tooth type 2. Clin. Genet. 82:157–164. 10.1111/j.1399-0004.2011.01717.x 21623771

[bib63] Cushion, T.D., A.R. Paciorkowski, D.T. Pilz, J.G. Mullins, L.E. Seltzer, R.W. Marion, E. Tuttle, D. Ghoneim, S.L. Christian, S.K. Chung, . 2014. De novo mutations in the beta-tubulin gene TUBB2A cause simplified gyral patterning and infantile-onset epilepsy. Am. J. Hum. Genet. 94:634–641. 10.1016/j.ajhg.2014.03.009 24702957 PMC3980418

[bib64] Dalla Costa, I., C.N. Buchanan, M.D. Zdradzinski, P.K. Sahoo, T.P. Smith, E. Thames, A.N. Kar, and J.L. Twiss. 2021. The functional organization of axonal mRNA transport and translation. Nat. Rev. Neurosci. 22:77–91. 10.1038/s41583-020-00407-7 33288912 PMC8161363

[bib65] de Boer, E.M.J., W. van Rheenen, H.S. Goedee, E.J. Kamsteeg, E.H. Brilstra, J.H. Veldink, L.H. van Den Berg, and M.A. van Es. 2021. Genotype-phenotype correlations of KIF5A stalk domain variants. Amyotroph. Lateral Scler. Frontotemporal Degener. 22:561–570. 10.1080/21678421.2021.1907412 33829936

[bib66] De Boulle, K., A.J.M.H. Verkerk, E. Reyniers, L. Vits, J. Hendrickx, B. Van Roy, F. Van den Bos, E. de Graaff, B.A. Oostra, and P.J. Willems. 1993. A point mutation in the FMR-1 gene associated with fragile X mental retardation. Nat. Genet. 3:31–35. 10.1038/ng0193-31 8490650

[bib67] de Ligt, J., M.H. Willemsen, B.W. van Bon, T. Kleefstra, H.G. Yntema, T. Kroes, A.T. Vulto-van Silfhout, D.A. Koolen, P. de Vries, C. Gilissen, . 2012. Diagnostic exome sequencing in persons with severe intellectual disability. N. Engl. J. Med. 367:1921–1929. 10.1056/NEJMoa1206524 23033978

[bib68] de Souza, P.V.S., W.B.V. de Rezende Pinto, G.N. de Rezende Batistella, T. Bortholin, and A.S.B. Oliveira. 2017. Hereditary spastic paraplegia: Clinical and genetic hallmarks. Cerebellum. 16:525–551. 10.1007/s12311-016-0803-z 27271711

[bib69] Deinhardt, K., S. Salinas, C. Verastegui, R. Watson, D. Worth, S. Hanrahan, C. Bucci, and G. Schiavo. 2006. Rab5 and Rab7 control endocytic sorting along the axonal retrograde transport pathway. Neuron. 52:293–305. 10.1016/j.neuron.2006.08.018 17046692

[bib70] Devine, M.J., and J.T. Kittler. 2018. Mitochondria at the neuronal presynapse in health and disease. Nat. Rev. Neurosci. 19:63–80. 10.1038/nrn.2017.170 29348666

[bib71] Di Giovanni, J., and Z.H. Sheng. 2015. Regulation of synaptic activity by snapin-mediated endolysosomal transport and sorting. EMBO J. 34:2059–2077. 10.15252/embj.201591125 26108535 PMC4551352

[bib72] Dolma, K., G.J. Iacobucci, K. Hong Zheng, J. Shandilya, E. Toska, J.A. White II, E. Spina, and S. Gunawardena. 2014. Presenilin influences glycogen synthase kinase-3 β (GSK-3β) for kinesin-1 and dynein function during axonal transport. Hum. Mol. Genet. 23:1121–1133. 10.1093/hmg/ddt505 24105467

[bib297] Dou, D., J. Aiken, and E.L.F. Holzbaur. 2024. RAB3 phosphorylation by pathogenic LRRK2 impairs trafficking of synaptic vesicle precursors. J. Cell Biol. 223:e202307092. 10.1083/jcb.202307092 38512027 PMC10959120

[bib74] Drévillon, L., A. Megarbane, B. Demeer, C. Matar, P. Benit, A. Briand-Suleau, V. Bodereau, J. Ghoumid, M. Nasser, X. Decrouy, . 2013. KBP-cytoskeleton interactions underlie developmental anomalies in Goldberg-Shprintzen syndrome. Hum. Mol. Genet. 22:2387–2399. 10.1093/hmg/ddt083 23427148

[bib75] Du, J., Y. Wei, L. Liu, Y. Wang, R. Khairova, R. Blumenthal, T. Tragon, J.G. Hunsberger, R. Machado-Vieira, W. Drevets, . 2010. A kinesin signaling complex mediates the ability of GSK-3beta to affect mood-associated behaviors. Proc. Natl. Acad. Sci. USA. 107:11573–11578. 10.1073/pnas.0913138107 20534517 PMC2895136

[bib76] Duis, J., S. Dean, C. Applegate, A. Harper, R. Xiao, W. He, J.D. Dollar, L.R. Sun, M.B. Waberski, T.O. Crawford, . 2016. KIF5A mutations cause an infantile onset phenotype including severe myoclonus with evidence of mitochondrial dysfunction. Ann. Neurol. 80:633–637. 10.1002/ana.24744 27463701 PMC5042851

[bib77] Edwards, S.L., N.K. Charlie, J.E. Richmond, J. Hegermann, S. Eimer, and K.G. Miller. 2009. Impaired dense core vesicle maturation in *Caenorhabditis elegans* mutants lacking Rab2. J. Cell Biol. 186:881–895. 10.1083/jcb.200902095 19797080 PMC2753164

[bib78] Ehinger, Y., J. Bruyère, N. Panayotis, Y.S. Abada, E. Borloz, V. Matagne, C. Scaramuzzino, H. Vitet, B. Delatour, L. Saidi, . 2020. Huntingtin phosphorylation governs BDNF homeostasis and improves the phenotype of Mecp2 knockout mice. EMBO Mol. Med. 12:e10889. 10.15252/emmm.201910889 31913581 PMC7005633

[bib79] Emperador-Melero, J., and P.S. Kaeser. 2020. Assembly of the presynaptic active zone. Curr. Opin. Neurobiol. 63:95–103. 10.1016/j.conb.2020.03.008 32403081 PMC7483790

[bib80] Engelender, S., A.H. Sharp, V. Colomer, M.K. Tokito, A. Lanahan, P. Worley, E.L. Holzbaur, and C.A. Ross. 1997. Huntingtin-associated protein 1 (HAP1) interacts with the p150Glued subunit of dynactin. Hum. Mol. Genet. 6:2205–2212. 10.1093/hmg/6.13.2205 9361024

[bib81] Exposito-Alonso, D., and B. Rico. 2022. Mechanisms underlying circuit dysfunction in neurodevelopmental disorders. Annu. Rev. Genet. 56:391–422. 10.1146/annurev-genet-072820-023642 36055969

[bib82] Fallet-Bianco, C., A. Laquerrière, K. Poirier, F. Razavi, F. Guimiot, P. Dias, L. Loeuillet, K. Lascelles, C. Beldjord, N. Carion, . 2014. Mutations in tubulin genes are frequent causes of various foetal malformations of cortical development including microlissencephaly. Acta Neuropathol. Commun. 2:69. 10.1186/2051-5960-2-69 25059107 PMC4222268

[bib83] Farfel-Becker, T., J.C. Roney, X.T. Cheng, S. Li, S.R. Cuddy, and Z.H. Sheng. 2019. Neuronal soma-derived degradative lysosomes are continuously delivered to distal axons to maintain local degradation capacity. Cell Rep. 28:51–64.e4. 10.1016/j.celrep.2019.06.013 31269450 PMC6696943

[bib84] Fazeli, W., P. Herkenrath, B. Stiller, A. Neugebauer, J. Fricke, R. Lang-Roth, G. Nürnberg, M. Thoenes, J. Becker, J. Altmüller, . 2017. A TUBB6 mutation is associated with autosomal dominant non-progressive congenital facial palsy, bilateral ptosis and velopharyngeal dysfunction. Hum. Mol. Genet. 26:4055–4066. 10.1093/hmg/ddx296 29016863

[bib85] Fejtova, A., D. Davydova, F. Bischof, V. Lazarevic, W.D. Altrock, S. Romorini, C. Schöne, W. Zuschratter, M.R. Kreutz, C.C. Garner, . 2009. Dynein light chain regulates axonal trafficking and synaptic levels of Bassoon. J. Cell Biol. 185:341–355. 10.1083/jcb.200807155 19380881 PMC2700376

[bib86] Flores, R. III, Y. Hirota, B. Armstrong, A. Sawa, and T. Tomoda. 2011. DISC1 regulates synaptic vesicle transport via a lithium-sensitive pathway. Neurosci. Res. 71:71–77. 10.1016/j.neures.2011.05.014 21664390 PMC3156137

[bib87] Fornasiero, E.F., S. Mandad, H. Wildhagen, M. Alevra, B. Rammner, S. Keihani, F. Opazo, I. Urban, T. Ischebeck, M.S. Sakib, . 2018. Precisely measured protein lifetimes in the mouse brain reveal differences across tissues and subcellular fractions. Nat. Commun. 9:4230. 10.1038/s41467-018-06519-0 30315172 PMC6185916

[bib88] Fourel, G., and C. Boscheron. 2020. Tubulin mutations in neurodevelopmental disorders as a tool to decipher microtubule function. FEBS Lett. 594:3409–3438. 10.1002/1873-3468.13958 33064843

[bib89] Friedman, H.V., T. Bresler, C.C. Garner, and N.E. Ziv. 2000. Assembly of new individual excitatory synapses: Time course and temporal order of synaptic molecule recruitment. Neuron. 27:57–69. 10.1016/s0896-6273(00)00009-x 10939331

[bib90] Fuchs, C., R. Rimondini, R. Viggiano, S. Trazzi, M. De Franceschi, R. Bartesaghi, and E. Ciani. 2015. Inhibition of GSK3β rescues hippocampal development and learning in a mouse model of CDKL5 disorder. Neurobiol. Dis. 82:298–310. 10.1016/j.nbd.2015.06.018 26143616

[bib91] Fukuda, Y., M.F. Pazyra-Murphy, E.S. Silagi, O.E. Tasdemir-Yilmaz, Y. Li, L. Rose, Z.C. Yeoh, N.E. Vangos, E.A. Geffken, H.S. Seo, . 2021. Binding and transport of SFPQ-RNA granules by KIF5A/KLC1 motors promotes axon survival. J. Cell Biol. 220:e202005051. 10.1083/jcb.202005051 33284322 PMC7721913

[bib295] Gambino, G., V. Rizzo, G. Giglia, G. Ferraro, and P. Sardo. 2022. Microtubule Dynamics and Neuronal Excitability: Advances on Cytoskeletal Components Implicated in Epileptic Phenomena. Cell Mol. Neurobiol. 42:533–543. 32929563 10.1007/s10571-020-00963-7 PMC8891195

[bib92] Gao, F.J., S. Hebbar, X.A. Gao, M. Alexander, J.P. Pandey, M.D. Walla, W.E. Cotham, S.J. King, and D.S. Smith. 2015. GSK-3β phosphorylation of cytoplasmic dynein reduces Ndel1 binding to intermediate chains and alters dynein motility. Traffic. 16:941–961. 10.1111/tra.12304 26010407 PMC4543430

[bib93] Garner, C.C., S. Kindler, and E.D. Gundelfinger. 2000. Molecular determinants of presynaptic active zones. Curr. Opin. Neurobiol. 10:321–327. 10.1016/s0959-4388(00)00093-3 10851173

[bib94] Garrott, S.R., J.P. Gillies, and M.E. DeSantis. 2022. Nde1 and Ndel1: Outstanding mysteries in dynein-mediated transport. Front. Cell Dev. Biol. 10:871935. 10.3389/fcell.2022.871935 35493069 PMC9041303

[bib95] Ge, W.R., P.P. Fu, W.N. Zhang, B. Zhang, Y.X. Ding, and G. Yang. 2023. Case report: Genotype and phenotype of DYNC1H1-related malformations of cortical development: A case report and literature review. Front. Neurol. 14:1163803. 10.3389/fneur.2023.1163803 37181555 PMC10167015

[bib96] Gershoni-Emek, N., T. Altman, A. Ionescu, C.J. Costa, T. Gradus-Pery, D.E. Willis, and E. Perlson. 2018. Localization of RNAi machinery to axonal branch points and growth cones is facilitated by mitochondria and is disrupted in ALS. Front. Mol. Neurosci. 11:311. 10.3389/fnmol.2018.00311 30233312 PMC6134038

[bib97] Gicking, A.M., T.C. Ma, Q. Feng, R. Jiang, S. Badieyan, M.A. Cianfrocco, and W.O. Hancock. 2022. Kinesin-1, -2, and -3 motors use family-specific mechanochemical strategies to effectively compete with dynein during bidirectional transport. Elife. 11:e82228. 10.7554/eLife.82228 36125250 PMC9545524

[bib98] Gill, S.R., T.A. Schroer, I. Szilak, E.R. Steuer, M.P. Sheetz, and D.W. Cleveland. 1991. Dynactin, a conserved, ubiquitously expressed component of an activator of vesicle motility mediated by cytoplasmic dynein. J. Cell Biol. 115:1639–1650. 10.1083/jcb.115.6.1639 1836789 PMC2289205

[bib99] Gold, W.A., T.A. Lacina, L.C. Cantrill, and J. Christodoulou. 2015. MeCP2 deficiency is associated with reduced levels of tubulin acetylation and can be restored using HDAC6 inhibitors. J. Mol. Med. 93:63–72. 10.1007/s00109-014-1202-x 25209898

[bib100] Goodwin, P.R., and P. Juo. 2013. The scaffolding protein SYD-2/Liprin-α regulates the mobility and polarized distribution of dense-core vesicles in *C. elegans* motor neurons. PLoS One. 8:e54763. 10.1371/journal.pone.0054763 23358451 PMC3554613

[bib101] Grant, S.G. 2012. Synaptopathies: Diseases of the synaptome. Curr. Opin. Neurobiol. 22:522–529. 10.1016/j.conb.2012.02.002 22409856

[bib102] Grigoriev, I., D. Splinter, N. Keijzer, P.S. Wulf, J. Demmers, T. Ohtsuka, M. Modesti, I.V. Maly, F. Grosveld, C.C. Hoogenraad, and A. Akhmanova. 2007. Rab6 regulates transport and targeting of exocytotic carriers. Dev. Cell. 13:305–314. 10.1016/j.devcel.2007.06.010 17681140

[bib103] Grønskov, K., K. Brøndum-Nielsen, A. Dedic, and H. Hjalgrim. 2011. A nonsense mutation in FMR1 causing fragile X syndrome. Eur. J. Hum. Genet. 19:489–491. 10.1038/ejhg.2010.223 21267007 PMC3060329

[bib104] Grossman, A.W., G.M. Aldridge, I.J. Weiler, and W.T. Greenough. 2006. Local protein synthesis and spine morphogenesis: Fragile X syndrome and beyond. J. Neurosci. 26:7151–7155. 10.1523/JNEUROSCI.1790-06.2006 16822971 PMC6673953

[bib105] Guedes-Dias, P., and E.L.F. Holzbaur. 2019. Axonal transport: Driving synaptic function. Science. 366:eaaw9997. 10.1126/science.aaw9997 31601744 PMC6996143

[bib106] Guedes-Dias, P., J.J. Nirschl, N. Abreu, M.K. Tokito, C. Janke, M.M. Magiera, and E.L.F. Holzbaur. 2019. Kinesin-3 responds to local microtubule dynamics to target synaptic cargo delivery to the presynapse. Curr. Biol. 29:268–282.e8. 10.1016/j.cub.2018.11.065 30612907 PMC6342647

[bib107] Guo, W., A.C. Murthy, L. Zhang, E.B. Johnson, E.G. Schaller, A.M. Allan, and X. Zhao. 2012. Inhibition of GSK3β improves hippocampus-dependent learning and rescues neurogenesis in a mouse model of fragile X syndrome. Hum. Mol. Genet. 21:681–691. 10.1093/hmg/ddr501 22048960 PMC3259018

[bib108] Gutnick, A., M.R. Banghart, E.R. West, and T.L. Schwarz. 2019. The light-sensitive dimerizer zapalog reveals distinct modes of immobilization for axonal mitochondria. Nat. Cell Biol. 21:768–777. 10.1038/s41556-019-0317-2 31061466 PMC6662610

[bib109] Guven, A., A. Gunduz, T.M. Bozoglu, C. Yalcinkaya, and A. Tolun. 2012. Novel NDE1 homozygous mutation resulting in microhydranencephaly and not microlyssencephaly. Neurogenetics. 13:189–194. 10.1007/s10048-012-0326-9 22526350

[bib110] Hafner, A.S., P.G. Donlin-Asp, B. Leitch, E. Herzog, and E.M. Schuman. 2019. Local protein synthesis is a ubiquitous feature of neuronal pre- and postsynaptic compartments. Science. 364:eaau3644. 10.1126/science.aau3644 31097639

[bib111] Hall, D.H., and E.M. Hedgecock. 1991. Kinesin-related gene unc-104 is required for axonal-transport of synaptic vesicles in C-elegans. Cell. 65:837–847. 10.1016/0092-8674(91)90391-b 1710172

[bib112] Hammond, J.W., D. Cai, T.L. Blasius, Z. Li, Y. Jiang, G.T. Jih, E. Meyhofer, and K.J. Verhey. 2009. Mammalian Kinesin-3 motors are dimeric in vivo and move by processive motility upon release of autoinhibition. PLoS Biol. 7:e72. 10.1371/journal.pbio.1000072 19338388 PMC2661964

[bib113] Hanson, J.E., and D.V. Madison. 2007. Presynaptic FMR1 genotype influences the degree of synaptic connectivity in a mosaic mouse model of fragile X syndrome. J. Neurosci. 27:4014–4018. 10.1523/JNEUROSCI.4717-06.2007 17428978 PMC6672544

[bib114] Harms, M.B., K.M. Ori-McKenney, M. Scoto, E.P. Tuck, S. Bell, D. Ma, S. Masi, P. Allred, M. Al-Lozi, M.M. Reilly, . 2012. Mutations in the tail domain of DYNC1H1 cause dominant spinal muscular atrophy. Neurology. 78:1714–1720. 10.1212/WNL.0b013e3182556c05 22459677 PMC3359582

[bib115] Harrington, A.W., and D.D. Ginty. 2013. Long-distance retrograde neurotrophic factor signalling in neurons. Nat. Rev. Neurosci. 14:177–187. 10.1038/nrn3253 23422909

[bib116] Heerssen, H.M., M.F. Pazyra, and R.A. Segal. 2004. Dynein motors transport activated Trks to promote survival of target-dependent neurons. Nat. Neurosci. 7:596–604. 10.1038/nn1242 15122257

[bib117] Herculano-Houzel, S. 2012. The remarkable, yet not extraordinary, human brain as a scaled-up primate brain and its associated cost. Proc. Natl. Acad. Sci. USA. 109:10661–10668. 10.1073/pnas.1201895109 22723358 PMC3386878

[bib118] Herman, G.E., W. Sadee, and E. Barrie. 2016. A collaborative translational autism research program for the military. https://apps.dtic.mil/sti/citations/ADA631878.

[bib119] Hirokawa, N., S. Niwa, and Y. Tanaka. 2010. Molecular motors in neurons: Transport mechanisms and roles in brain function, development, and disease. Neuron. 68:610–638. 10.1016/j.neuron.2010.09.039 21092854

[bib120] Hirotsune, S., M.W. Fleck, M.J. Gambello, G.J. Bix, A. Chen, G.D. Clark, D.H. Ledbetter, C.J. McBain, and A. Wynshaw-Boris. 1998. Graded reduction of Pafah1b1 (Lis1) activity results in neuronal migration defects and early embryonic lethality. Nat. Genet. 19:333–339. 10.1038/1221 9697693

[bib121] Hoang, H.T., M.A. Schlager, A.P. Carter, and S.L. Bullock. 2017. DYNC1H1 mutations associated with neurological diseases compromise processivity of dynein-dynactin-cargo adaptor complexes. Proc. Natl. Acad. Sci. USA. 114:E1597–E1606. 10.1073/pnas.1620141114 28196890 PMC5338514

[bib122] Holderith, N., A. Lorincz, G. Katona, B. Rózsa, A. Kulik, M. Watanabe, and Z. Nusser. 2012. Release probability of hippocampal glutamatergic terminals scales with the size of the active zone. Nat. Neurosci. 15:988–997. 10.1038/nn.3137 22683683 PMC3386897

[bib292] Hoogenraad, C.C., and F. Bradke. 2009. Control of neuronal polarity and plasticity--a renaissance for microtubules? Trends Cell Biol. 19(12):669–676. 10.1016/j.tcb.2009.08.006 19801190

[bib123] Htet, Z.M., J.P. Gillies, R.W. Baker, A.E. Leschziner, M.E. DeSantis, and S.L. Reck-Peterson. 2020. LIS1 promotes the formation of activated cytoplasmic dynein-1 complexes. Nat. Cell Biol. 22:518–525. 10.1038/s41556-020-0506-z 32341549 PMC7271980

[bib124] Huang, S.H., S. Duan, T. Sun, J. Wang, L. Zhao, Z. Geng, J. Yan, H.J. Sun, and Z.Y. Chen. 2011. JIP3 mediates TrkB axonal anterograde transport and enhances BDNF signaling by directly bridging TrkB with kinesin-1. J. Neurosci. 31:10602–10614. 10.1523/JNEUROSCI.0436-11.2011 21775604 PMC6622626

[bib125] Huang, Y., J. Jiao, M. Zhang, M. Situ, D. Yuan, P. Lyu, S. Li, Z. Wang, Y. Yang, and Y. Huang. 2021. [A study on KIF1A gene missense variant analysis and its protein expression and structure profiles of an autism spectrum disorder family trio]. Zhonghua Yi Xue Yi Chuan Xue Za Zhi. 38:620–625. 10.3760/cma.j.cn511374-20210120-00060 34247363

[bib126] Huynh, W., and R.D. Vale. 2017. Disease-associated mutations in human BICD2 hyperactivate motility of dynein-dynactin. J. Cell Biol. 216:3051–3060. 10.1083/jcb.201703201 28883039 PMC5626548

[bib127] Ishizuka, N., J. Weber, and D.G. Amaral. 1990. Organization of intrahippocampal projections originating from CA3 pyramidal cells in the rat. J. Comp. Neurol. 295:580–623. 10.1002/cne.902950407 2358523

[bib128] Isobe, K., D. Ieda, F. Miya, R. Miyachi, S. Otsuji, M. Asai, T. Tsunoda, K. Kosaki, A. Hattori, S. Saitoh, and M. Mizuno. 2022. Hemorrhagic shock and encephalopathy syndrome in a patient with a de novo heterozygous variant in KIF1A. Brain Dev. 44:249–253. 10.1016/j.braindev.2021.11.007 34916088

[bib129] Jamuar, S.S., C.A. Walsh, M. Kircher, A.M. D’Gama, J. Wang, B.J. Barry, X. Zhang, R.S. Hill, . 2014. Somatic mutations in cerebral cortical malformations. N. Engl. J. Med. 371:2038. 10.1056/NEJMc1411784 PMC4274952 25140959

[bib130] Jin, Y., and C.C. Garner. 2008. Molecular mechanisms of presynaptic differentiation. Annu. Rev. Cell Dev. Biol. 24:237–262. 10.1146/annurev.cellbio.23.090506.123417 18588488

[bib131] Jin, E.J., F.R. Kiral, M.N. Ozel, L.S. Burchardt, M. Osterland, D. Epstein, H. Wolfenberg, S. Prohaska, and P.R. Hiesinger. 2018. Live observation of two parallel membrane degradation pathways at axon terminals. Curr. Biol. 28:1027–1038.e4. 10.1016/j.cub.2018.02.032 29551411 PMC5944365

[bib132] Kanai, Y., N. Dohmae, and N. Hirokawa. 2004. Kinesin transports RNA: Isolation and characterization of an RNA-transporting granule. Neuron. 43:513–525. 10.1016/j.neuron.2004.07.022 15312650

[bib133] Kapitein, L.C., M.A. Schlager, M. Kuijpers, P.S. Wulf, M. van Spronsen, F.C. MacKintosh, and C.C. Hoogenraad. 2010. Mixed microtubules steer dynein-driven cargo transport into dendrites. Curr. Biol. 20:290–299. 10.1016/j.cub.2009.12.052 20137950

[bib134] Karasmanis, E.P., J.M. Reimer, A.A. Kendrick, K.H.V. Nguyen, J.A. Rodriguez, J.B. Truong, I. Lahiri, S.L. Reck-Peterson, and A.E. Leschziner. 2023. Lis1 relieves cytoplasmic dynein-1 autoinhibition by acting as a molecular wedge. Nat. Struct. Mol. Biol. 30:1357–1364. 10.1038/s41594-023-01069-6 37620585 PMC10497415

[bib135] Kevenaar, J.T., S. Bianchi, M. van Spronsen, N. Olieric, J. Lipka, C.P. Frias, M. Mikhaylova, M. Harterink, N. Keijzer, P.S. Wulf, . 2016. Kinesin-binding protein controls microtubule dynamics and cargo trafficking by regulating kinesin motor activity. Curr. Biol. 26:849–861. 10.1016/j.cub.2016.01.048 26948876

[bib136] Kimura, H., D. Tsuboi, C. Wang, I. Kushima, T. Koide, M. Ikeda, Y. Iwayama, T. Toyota, N. Yamamoto, S. Kunimoto, . 2015. Identification of rare, single-nucleotide mutations in NDE1 and their contributions to schizophrenia susceptibility. Schizophr. Bull. 41:744–753. 10.1093/schbul/sbu147 25332407 PMC4393687

[bib137] King, S.J., and T.A. Schroer. 2000. Dynactin increases the processivity of the cytoplasmic dynein motor. Nat. Cell Biol. 2:20–24. 10.1038/71338 10620802

[bib138] Klassen, M.P., Y.E. Wu, C.I. Maeder, I. Nakae, J.G. Cueva, E.K. Lehrman, M. Tada, K. Gengyo-Ando, G.J. Wang, M. Goodman, . 2010. An Arf-like small G protein, ARL-8, promotes the axonal transport of presynaptic cargoes by suppressing vesicle aggregation. Neuron. 66:710–723. 10.1016/j.neuron.2010.04.033 20547129 PMC3168544

[bib139] Klebe, S., A. Lossos, H. Azzedine, E. Mundwiller, R. Sheffer, M. Gaussen, C. Marelli, M. Nawara, W. Carpentier, V. Meyer, . 2012. KIF1A missense mutations in SPG30, an autosomal recessive spastic paraplegia: Distinct phenotypes according to the nature of the mutations. Eur. J. Hum. Genet. 20:645–649. 10.1038/ejhg.2011.261 22258533 PMC3355258

[bib140] Klopfenstein, D.R., and R.D. Vale. 2004. The lipid binding pleckstrin homology domain in UNC-104 kinesin is necessary for synaptic vesicle transport in *Caenorhabditis elegans*. Mol. Biol. Cell. 15:3729–3739. 10.1091/mbc.e04-04-0326 15155810 PMC491832

[bib141] Koboldt, D.C., R.D. Kastury, M.A. Waldrop, B.J. Kelly, T.M. Mosher, H. McLaughlin, D. Corsmeier, J.L. Slaughter, K.M. Flanigan, K.L. McBride, . 2018. In-frame de novo mutation in BICD2 in two patients with muscular atrophy and arthrogryposis. Cold Spring Harb. Mol. Case Stud. 4:a003160. 10.1101/mcs.a003160 30054298 PMC6169820

[bib142] Kondo, S., R. Sato-Yoshitake, Y. Noda, H. Aizawa, T. Nakata, Y. Matsuura, and N. Hirokawa. 1994. KIF3A is a new microtubule-based anterograde motor in the nerve axon. J. Cell Biol. 125:1095–1107. 10.1083/jcb.125.5.1095 7515068 PMC2120052

[bib143] Kondo, M., Y. Takei, and N. Hirokawa. 2012. Motor protein KIF1A is essential for hippocampal synaptogenesis and learning enhancement in an enriched environment. Neuron. 73:743–757. 10.1016/j.neuron.2011.12.020 22365548

[bib144] Konishi, Y., and M. Setou. 2009. Tubulin tyrosination navigates the kinesin-1 motor domain to axons. Nat. Neurosci. 12:559–567. 10.1038/nn.2314 19377471

[bib291] Kononenko, N.L., G.A. Classen, M. Kuijpers, D. Puchkov, T. Maritzen, . 2017. Retrograde transport of TrkB-containing autophagosomes via the adaptor AP-2 mediates neuronal complexity and prevents neurodegeneration. Nat. Commun. 8:14819. 10.1038/ncomms14819 28387218 PMC5385568

[bib145] Kremer, E.J., M. Pritchard, M. Lynch, S. Yu, K. Holman, E. Baker, S.T. Warren, D. Schlessinger, G.R. Sutherland, and R.I. Richards. 1991. Mapping of DNA instability at the fragile X to a trinucleotide repeat sequence p(CCG)n. Science. 252:1711–1714. 10.1126/science.1675488 1675488

[bib146] Kurihara, M., H. Ishiura, T. Bannai, J. Mitsui, J. Yoshimura, S. Morishita, T. Hayashi, J. Shimizu, T. Toda, and S. Tsuji. 2020. A Novel de novo KIF1A Mutation in a Patient with Autism, Hyperactivity, Epilepsy, Sensory Disturbance, and Spastic Paraplegia. Intern. Med. 59:839–842. 10.2169/internalmedicine.3661-19 31813911 PMC7118386

[bib147] Kwinter, D.M., K. Lo, P. Mafi, and M.A. Silverman. 2009. Dynactin regulates bidirectional transport of dense-core vesicles in the axon and dendrites of cultured hippocampal neurons. Neuroscience. 162:1001–1010. 10.1016/j.neuroscience.2009.05.038 19497353

[bib148] Laquerriere, A., M. Gonzales, Y. Saillour, M. Cavallin, N. Joyē, C. Quēlin, L. Bidat, M. Dommergues, G. Plessis, F. Encha-Razavi, . 2016. De novo TUBB2B mutation causes fetal akinesia deformation sequence with microlissencephaly: An unusual presentation of tubulinopathy. Eur. J. Med. Genet. 59:249–256. 10.1016/j.ejmg.2015.12.007 26732629

[bib149] Larti, F., K. Kahrizi, L. Musante, H. Hu, E. Papari, Z. Fattahi, N. Bazazzadegan, Z. Liu, M. Banan, M. Garshasbi, . 2015. A defect in the CLIP1 gene (CLIP-170) can cause autosomal recessive intellectual disability. Eur. J. Hum. Genet. 23:331–336. 10.1038/ejhg.2014.13 24569606 PMC4326716

[bib150] Lasser, M., J. Tiber, and L.A. Lowery. 2018. The role of the microtubule cytoskeleton in neurodevelopmental disorders. Front. Cell. Neurosci. 12:165. 10.3389/fncel.2018.00165 29962938 PMC6010848

[bib151] Lee, J.R., M. Srour, D. Kim, F.F. Hamdan, S.H. Lim, C. Brunel-Guitton, J.C. Décarie, E. Rossignol, G.A. Mitchell, A. Schreiber, . 2015. De novo mutations in the motor domain of KIF1A cause cognitive impairment, spastic paraparesis, axonal neuropathy, and cerebellar atrophy. Hum. Mutat. 36:69–78. 10.1002/humu.22709 25265257

[bib152] Lee, S.J., J.A. Oses-Prieto, R. Kawaguchi, P.K. Sahoo, A.N. Kar, M. Rozenbaum, D. Oliver, S. Chand, H. Ji, M. Shtutman, . 2018. hnRNPs interacting with mRNA localization motifs define axonal RNA regulons. Mol. Cell. Proteomics. 17:2091–2106. 10.1074/mcp.RA118.000603 30038033 PMC6210225

[bib153] Li, S., and Z.H. Sheng. 2022. Energy matters: Presynaptic metabolism and the maintenance of synaptic transmission. Nat. Rev. Neurosci. 23:4–22. 10.1038/s41583-021-00535-8 34782781

[bib154] Li, X.G., P. Somogyi, A. Ylinen, and G. Buzsáki. 1994. The hippocampal CA3 network: An in vivo intracellular labeling study. J. Comp. Neurol. 339:181–208. 10.1002/cne.903390204 8300905

[bib155] Li, S., G.J. Xiong, N. Huang, and Z.H. Sheng. 2020. The cross-talk of energy sensing and mitochondrial anchoring sustains synaptic efficacy by maintaining presynaptic metabolism. Nat. Metab. 2:1077–1095. 10.1038/s42255-020-00289-0 33020662 PMC7572785

[bib156] Liaci, C., M. Camera, G. Caslini, S. Rando, S. Contino, V. Romano, and G.R. Merlo. 2021. Neuronal cytoskeleton in intellectual disability: From systems biology and modeling to therapeutic opportunities. Int. J. Mol. Sci. 22:6167. 10.3390/ijms22116167 34200511 PMC8201358

[bib157] Liao, Y.C., M.S. Fernandopulle, G. Wang, H. Choi, L. Hao, C.M. Drerup, R. Patel, S. Qamar, J. Nixon-Abell, Y. Shen, . 2019. RNA granules hitchhike on lysosomes for long-distance transport, using Annexin A11 as a molecular tether. Cell. 179:147–164.e20. 10.1016/j.cell.2019.08.050 31539493 PMC6890474

[bib158] Lim, A., A. Rechtsteiner, and W.M. Saxton. 2017. Two kinesins drive anterograde neuropeptide transport. Mol. Biol. Cell. 28:3542–3553. 10.1091/mbc.e16-12-0820 28904207 PMC5683764

[bib159] Lim, Y., L.L. Wu, S. Chen, Y. Sun, S.L. Vijayaraj, M. Yang, L. Bobrovskaya, D. Keating, X.J. Li, and X.F. Zhou. 2018. HAP1 is required for endocytosis and signalling of BDNF and its receptors in neurons. Mol. Neurobiol. 55:1815–1830. 10.1007/s12035-016-0379-0 28083816 PMC5821608

[bib160] Lipka, J., M. Kuijpers, J. Jaworski, and C.C. Hoogenraad. 2013. Mutations in cytoplasmic dynein and its regulators cause malformations of cortical development and neurodegenerative diseases. Biochem. Soc. Trans. 41:1605–1612. 10.1042/BST20130188 24256262

[bib161] Lipska, B.K., T. Peters, T.M. Hyde, N. Halim, C. Horowitz, S. Mitkus, C.S. Weickert, M. Matsumoto, A. Sawa, R.E. Straub, . 2006. Expression of DISC1 binding partners is reduced in schizophrenia and associated with DISC1 SNPs. Hum. Mol. Genet. 15:1245–1258. 10.1093/hmg/ddl040 16510495

[bib162] Lipton, D.M., C.I. Maeder, and K. Shen. 2018. Rapid assembly of presynaptic materials behind the growth cone in dopaminergic neurons is mediated by precise regulation of axonal transport. Cell Rep. 24:2709–2722. 10.1016/j.celrep.2018.07.096 30184504 PMC6179448

[bib163] Lo, K.Y., A. Kuzmin, S.M. Unger, J.D. Petersen, and M.A. Silverman. 2011. KIF1A is the primary anterograde motor protein required for the axonal transport of dense-core vesicles in cultured hippocampal neurons. Neurosci. Lett. 491:168–173. 10.1016/j.neulet.2011.01.018 21256924

[bib164] Lund, V.K., M.D. Lycas, A. Schack, R.C. Andersen, U. Gether, and O. Kjaerulff. 2021. Rab2 drives axonal transport of dense core vesicles and lysosomal organelles. Cell Rep. 35:108973. 10.1016/j.celrep.2021.108973 33852866

[bib165] Maas, C., V.I. Torres, W.D. Altrock, S. Leal-Ortiz, D. Wagh, R.T. Terry-Lorenzo, A. Fejtova, E.D. Gundelfinger, N.E. Ziv, and C.C. Garner. 2012. Formation of Golgi-derived active zone precursor vesicles. J. Neurosci. 32:11095–11108. 10.1523/JNEUROSCI.0195-12.2012 22875941 PMC3752076

[bib166] Macabuag, N., and A.C. Dolphin. 2015. Alternative splicing in Ca(V)2.2 regulates neuronal trafficking via adaptor protein complex-1 adaptor protein motifs. J. Neurosci. 35:14636–14652. 10.1523/JNEUROSCI.3034-15.2015 26511252 PMC4623230

[bib167] Mao, Y., X. Ge, C.L. Frank, J.M. Madison, A.N. Koehler, M.K. Doud, C. Tassa, E.M. Berry, T. Soda, K.K. Singh, . 2009. Disrupted in schizophrenia 1 regulates neuronal progenitor proliferation via modulation of GSK3beta/beta-catenin signaling. Cell. 136:1017–1031. 10.1016/j.cell.2008.12.044 19303846 PMC2704382

[bib169] Megagiannis, P., R. Suresh, G.A. Rouleau, and Y. Zhou. 2022. Reversibility and therapeutic development for neurodevelopmental disorders, insights from genetic animal models. Adv. Drug Deliv. Rev. 191:114562. 10.1016/j.addr.2022.114562 36183904

[bib170] Méreaux, J.L., G. Banneau, M. Papin, G. Coarelli, R. Valter, L. Raymond, B. Kol, O. Ariste, L. Parodi, L. Tissier, . 2022. Clinical and genetic spectra of 1550 index patients with hereditary spastic paraplegia. Brain. 145:1029–1037. 10.1093/brain/awab386 34983064

[bib171] Merianda, T.T., C. Gomes, S. Yoo, D. Vuppalanchi, and J.L. Twiss. 2013. Axonal localization of neuritin/CPG15 mRNA in neuronal populations through distinct 5′ and 3′ UTR elements. J. Neurosci. 33:13735–13742. 10.1523/JNEUROSCI.0962-13.2013 23966695 PMC3755718

[bib172] Michetti, C., A. Falace, F. Benfenati, and A. Fassio. 2022. Synaptic genes and neurodevelopmental disorders: From molecular mechanisms to developmental strategies of behavioral testing. Neurobiol. Dis. 173:105856. 10.1016/j.nbd.2022.105856 36070836

[bib173] Miller, K.E., J. DeProto, N. Kaufmann, B.N. Patel, A. Duckworth, and D. Van Vactor. 2005. Direct observation demonstrates that Liprin-alpha is required for trafficking of synaptic vesicles. Curr. Biol. 15:684–689. 10.1016/j.cub.2005.02.061 15823543

[bib174] Minoura, I., H. Takazaki, R. Ayukawa, C. Saruta, Y. Hachikubo, S. Uchimura, T. Hida, H. Kamiguchi, T. Shimogori, and E. Muto. 2016. Reversal of axonal growth defects in an extraocular fibrosis model by engineering the kinesin-microtubule interface. Nat. Commun. 7:10058. 10.1038/ncomms10058 26775887 PMC4735607

[bib175] Miryala, C.S.J., E.D. Holland, and E.W. Dent. 2022. Contributions of microtubule dynamics and transport to presynaptic and postsynaptic functions. Mol. Cell. Neurosci. 123:103787. 10.1016/j.mcn.2022.103787 36252720 PMC9838116

[bib176] Misgeld, T., and T.L. Schwarz. 2017. Mitostasis in neurons: Maintaining mitochondria in an extended cellular architecture. Neuron. 96:651–666. 10.1016/j.neuron.2017.09.055 29096078 PMC5687842

[bib177] Monroy, B.Y., D.L. Sawyer, B.E. Ackermann, M.M. Borden, T.C. Tan, and K.M. Ori-McKenney. 2018. Competition between microtubule-associated proteins directs motor transport. Nat. Commun. 9:1487. 10.1038/s41467-018-03909-2 29662074 PMC5902456

[bib178] Morciano, M., J. Burré, C. Corvey, M. Karas, H. Zimmermann, and W. Volknandt. 2005. Immunoisolation of two synaptic vesicle pools from synaptosomes: A proteomics analysis. J. Neurochem. 95:1732–1745. 10.1111/j.1471-4159.2005.03506.x 16269012

[bib179] Morikawa, M., N.U. Jerath, T. Ogawa, M. Morikawa, Y. Tanaka, M.E. Shy, S. Zuchner, and N. Hirokawa. 2022. A neuropathy-associated kinesin KIF1A mutation hyper-stabilizes the motor-neck interaction during the ATPase cycle. EMBO J. 41:e108899. 10.15252/embj.2021108899 35132656 PMC8886545

[bib180] Moutin, M.J., C. Bosc, L. Peris, and A. Andrieux. 2021. Tubulin post-translational modifications control neuronal development and functions. Dev. Neurobiol. 81:253–272. 10.1002/dneu.22774 33325152 PMC8246997

[bib181] Moya-Alvarado, G., M.V. Guerra, R. Tiburcio, E. Bravo, and F.C. Bronfman. 2022. The Rab11-regulated endocytic pathway and BDNF/TrkB signaling: Roles in plasticity changes and neurodegenerative diseases. Neurobiol. Dis. 171:105796. 10.1016/j.nbd.2022.105796 35728773

[bib293] Nair, A., A. Greeny, R. Rajendran, M.A. Abdelgawad, M.M. Ghoneim, . 2023. KIF1A-Associated Neurological Disorder: An Overview of a Rare Mutational Disease. Pharmaceuticals (Basel). 16:147. 10.3390/ph16020147 37259299 PMC9962247

[bib182] Nakata, T., S. Niwa, Y. Okada, F. Perez, and N. Hirokawa. 2011. Preferential binding of a kinesin-1 motor to GTP-tubulin-rich microtubules underlies polarized vesicle transport. J. Cell Biol. 194:245–255. 10.1083/jcb.201104034 21768290 PMC3144414

[bib183] Nassal, J.P., F.H. Murphy, R.F. Toonen, and M. Verhage. 2022. Differential axonal trafficking of Neuropeptide Y-, LAMP1-, and RAB7-tagged organelles in vivo. Elife. 11:e81721. 10.7554/eLife.81721 36459486 PMC9718525

[bib184] Nemani, T., D. Steel, M. Kaliakatsos, C. DeVile, A. Ververi, R. Scott, S. Getov, S. Sudhakar, A. Male, K. Mankad, . 2020. KIF1A-related disorders in children: A wide spectrum of central and peripheral nervous system involvement. J. Peripher. Nerv. Syst. 25:117–124. 10.1111/jns.12368 32096284

[bib185] Neveling, K., L.A. Martinez-Carrera, I. Hölker, A. Heister, A. Verrips, S.M. Hosseini-Barkooie, C. Gilissen, S. Vermeer, M. Pennings, R. Meijer, . 2013. Mutations in BICD2, which encodes a golgin and important motor adaptor, cause congenital autosomal-dominant spinal muscular atrophy. Am. J. Hum. Genet. 92:946–954. 10.1016/j.ajhg.2013.04.011 23664116 PMC3675237

[bib186] Nirschl, J.J., M.M. Magiera, J.E. Lazarus, C. Janke, and E.L. Holzbaur. 2016. α-Tubulin tyrosination and CLIP-170 phosphorylation regulate the initiation of dynein-driven transport in neurons. Cell Rep. 14:2637–2652. 10.1016/j.celrep.2016.02.046 26972003 PMC4819336

[bib187] Niwa, S., Y. Tanaka, and N. Hirokawa. 2008. KIF1Bbeta- and KIF1A-mediated axonal transport of presynaptic regulator Rab3 occurs in a GTP-dependent manner through DENN/MADD. Nat. Cell Biol. 10:1269–1279. 10.1038/ncb1785 18849981

[bib188] Niwa, S., H. Takahashi, and N. Hirokawa. 2013. β-Tubulin mutations that cause severe neuropathies disrupt axonal transport. EMBO J. 32:1352–1364. 10.1038/emboj.2013.59 23503589 PMC3655465

[bib189] Niwa, S., D.M. Lipton, M. Morikawa, C. Zhao, N. Hirokawa, H. Lu, and K. Shen. 2016. Autoinhibition of a neuronal kinesin UNC-104/KIF1A regulates the size and density of synapses. Cell Rep. 16:2129–2141. 10.1016/j.celrep.2016.07.043 27524618 PMC5432123

[bib190] Ohba, C., K. Haginoya, H. Osaka, K. Kubota, A. Ishiyama, T. Hiraide, H. Komaki, M. Sasaki, S. Miyatake, M. Nakashima, . 2015. De novo KIF1A mutations cause intellectual deficit, cerebellar atrophy, lower limb spasticity and visual disturbance. J. Hum. Genet. 60:739–742. 10.1038/jhg.2015.108 26354034

[bib191] Okada, Y., H. Yamazaki, Y. Sekine-Aizawa, and N. Hirokawa. 1995. The neuron-specific kinesin superfamily protein KIF1A is a unique monomeric motor for anterograde axonal transport of synaptic vesicle precursors. Cell. 81:769–780. 10.1016/0092-8674(95)90538-3 7539720

[bib192] Olenick, M.A., M. Tokito, M. Boczkowska, R. Dominguez, and E.L.F. Holzbaur. 2016. Hook adaptors induce unidirectional processive motility by enhancing the dynein-dynactin interaction. J. Biol. Chem. 291:18239–18251. 10.1074/jbc.M116.738211 27365401 PMC5000072

[bib193] Olenick, M.A., R. Dominguez, and E.L.F. Holzbaur. 2019. Dynein activator Hook1 is required for trafficking of BDNF-signaling endosomes in neurons. J. Cell Biol. 218:220–233. 10.1083/jcb.201805016 30373907 PMC6314548

[bib194] Ozlu, C., R.M. Bailey, S. Sinnett, and K.D. Goodspeed. 2021. Gene transfer therapy for neurodevelopmental disorders. Dev. Neurosci. 43:230–240. 10.1159/000515434 33882495

[bib195] O’Donnell, W.T., and S.T. Warren. 2002. A decade of molecular studies of fragile X syndrome. Annu. Rev. Neurosci. 25:315–338. 10.1146/annurev.neuro.25.112701.142909 12052912

[bib196] Pack-Chung, E., P.T. Kurshan, D.K. Dickman, and T.L. Schwarz. 2007. A Drosophila kinesin required for synaptic bouton formation and synaptic vesicle transport. Nat. Neurosci. 10:980–989. 10.1038/nn1936 17643120

[bib197] Parenti, I., L.G. Rabaneda, H. Schoen, and G. Novarino. 2020. Neurodevelopmental disorders: From genetics to functional pathways. Trends Neurosci. 43:608–621. 10.1016/j.tins.2020.05.004 32507511

[bib198] Peeters, K., I. Litvinenko, B. Asselbergh, L. Almeida-Souza, T. Chamova, T. Geuens, E. Ydens, M. Zimoń, J. Irobi, E. De Vriendt, . 2013. Molecular defects in the motor adaptor BICD2 cause proximal spinal muscular atrophy with autosomal-dominant inheritance. Am. J. Hum. Genet. 92:955–964. 10.1016/j.ajhg.2013.04.013 23664119 PMC3675262

[bib199] Pennings, M., M.I. Schouten, J. van Gaalen, R.P.P. Meijer, S.T. de Bot, M. Kriek, C.G.J. Saris, L.H. van den Berg, M.A. van Es, D.M.H. Zuidgeest, . 2020. KIF1A variants are a frequent cause of autosomal dominant hereditary spastic paraplegia. Eur. J. Hum. Genet. 28:40–49. 10.1038/s41431-019-0497-z 31488895 PMC6906463

[bib200] Petanjek, Z., M. Judaš, G. Šimic, M.R. Rasin, H.B. Uylings, P. Rakic, and I. Kostovic. 2011. Extraordinary neoteny of synaptic spines in the human prefrontal cortex. Proc. Natl. Acad. Sci. USA. 108:13281–13286. 10.1073/pnas.1105108108 21788513 PMC3156171

[bib201] Pham, C.L., and N.S. Morrissette. 2019. The tubulin mutation database: A resource for the cytoskeleton community. Cytoskeleton. 76:186–191. 10.1002/cm.21514 30667171

[bib202] Pichon, X., K. Moissoglu, E. Coleno, T. Wang, A. Imbert, M.C. Robert, M. Peter, R. Chouaib, T. Walter, F. Mueller, . 2021. The kinesin KIF1C transports APC-dependent mRNAs to cell protrusions. RNA. 27:1528–1544. 10.1261/rna.078576.120 34493599 PMC8594469

[bib203] Pilling, A.D., D. Horiuchi, C.M. Lively, and W.M. Saxton. 2006. Kinesin-1 and Dynein are the primary motors for fast transport of mitochondria in *Drosophila* motor axons. Mol. Biol. Cell. 17:2057–2068. 10.1091/mbc.e05-06-0526 16467387 PMC1415296

[bib204] Poirier, K., Y. Saillour, N. Bahi-Buisson, X.H. Jaglin, C. Fallet-Bianco, R. Nabbout, L. Castelnau-Ptakhine, A. Roubertie, T. Attie-Bitach, I. Desguerre, . 2010. Mutations in the neuronal ß-tubulin subunit TUBB3 result in malformation of cortical development and neuronal migration defects. Hum. Mol. Genet. 19:4462–4473. 10.1093/hmg/ddq377 20829227 PMC3298850

[bib205] Poirier, K., N. Lebrun, L. Broix, G. Tian, Y. Saillour, C. Boscheron, E. Parrini, S. Valence, B.S. Pierre, M. Oger, . 2013. Mutations in TUBG1, DYNC1H1, KIF5C and KIF2A cause malformations of cortical development and microcephaly. Nat. Genet. 45:639–647. 10.1038/ng.2613 23603762 PMC3826256

[bib206] Ravenscroft, G., N. Di Donato, G. Hahn, M.R. Davis, P.D. Craven, G. Poke, K.R. Neas, T.M. Neuhann, W.B. Dobyns, and N.G. Laing. 2016. Recurrent de novo BICD2 mutation associated with arthrogryposis multiplex congenita and bilateral perisylvian polymicrogyria. Neuromuscul. Disord. 26:744–748. 10.1016/j.nmd.2016.09.009 27751653

[bib207] Reck-Peterson, S.L., W.B. Redwine, R.D. Vale, and A.P. Carter. 2018. The cytoplasmic dynein transport machinery and its many cargoes. Nat. Rev. Mol. Cell Biol. 19:382–398. 10.1038/s41580-018-0004-3 29662141 PMC6457270

[bib208] Reiner, O., R. Carrozzo, Y. Shen, M. Wehnert, F. Faustinella, W.B. Dobyns, C.T. Caskey, and D.H. Ledbetter. 1993. Isolation of a Miller-Dieker lissencephaly gene containing G protein beta-subunit-like repeats. Nature. 364:717–721. 10.1038/364717a0 8355785

[bib209] Rivière, J.B., S. Ramalingam, V. Lavastre, M. Shekarabi, S. Holbert, J. Lafontaine, M. Srour, N. Merner, D. Rochefort, P. Hince, . 2011. KIF1A, an axonal transporter of synaptic vesicles, is mutated in hereditary sensory and autonomic neuropathy type 2. Am. J. Hum. Genet. 89:219–230. 10.1016/j.ajhg.2011.06.013 21820098 PMC3155159

[bib210] Rizalar, F.S., D.A. Roosen, and V. Haucke. 2021. A presynaptic perspective on transport and assembly mechanisms for synapse formation. Neuron. 109:27–41. 10.1016/j.neuron.2020.09.038 33098763

[bib211] Rodan, L.H., C.M. El Achkar, G.T. Berry, A. Poduri, S.P. Prabhu, E. Yang, and I. Anselm. 2017. De novo TUBB2A variant presenting with anterior temporal pachygyria. J. Child Neurol. 32:127–131. 10.1177/0883073816672998 27770045

[bib212] Roney, J.C., X.T. Cheng, and Z.H. Sheng. 2022. Neuronal endolysosomal transport and lysosomal functionality in maintaining axonostasis. J. Cell Biol. 221:e202111077. 10.1083/jcb.202111077 35142819 PMC8932522

[bib213] Rossor, A.M., E.C. Oates, H.K. Salter, Y. Liu, S.M. Murphy, R. Schule, M.A. Gonzalez, M. Scoto, R. Phadke, C.A. Sewry, . 2015. Phenotypic and molecular insights into spinal muscular atrophy due to mutations in BICD2. Brain. 138:293–310. 10.1093/brain/awu356 25497877 PMC4306822

[bib214] Rydzanicz, M., M. Jagła, J. Kosinska, T. Tomasik, A. Sobczak, A. Pollak, I. Herman-Sucharska, A. Walczak, P. Kwinta, and R. Płoski. 2017. KIF5A de novo mutation associated with myoclonic seizures and neonatal onset progressive leukoencephalopathy. Clin. Genet. 91:769–773. 10.1111/cge.12831 27414745

[bib215] Saillour, Y., N. Carion, C. Quelin, P.L. Leger, N. Boddaert, C. Elie, A. Toutain, S. Mercier, M.A. Barthez, M. Milh, . 2009. LIS1-related isolated lissencephaly: Spectrum of mutations and relationships with malformation severity. Arch. Neurol. 66:1007–1015. 10.1001/archneurol.2009.149 19667223

[bib294] Salehpour, S., F. Hashemi-Gorji, Z. Soltani, S. Ghafouri-Fard, and M. Miryounesi. 2017. Association of a Novel Nonsense Mutation in KIAA1279 with Goldberg-Shprintzen Syndrome. Iran J. Child Neurol. 11:70–74. 28277559 PMC5329763

[bib216] Sánchez Delgado, M., C. Camprubí, Z. Tümer, F. Martínez, M. Milà, and D. Monk. 2014. Screening individuals with intellectual disability, autism and Tourette’s syndrome for KCNK9 mutations and aberrant DNA methylation within the 8q24 imprinted cluster. Am. J. Med. Genet. B. Neuropsychiatr. Genet. 165B:472–478. 10.1002/ajmg.b.32250 24980697

[bib217] Scaramuzzino, C., E.C. Cuoc, P. Pla, S. Humbert, and F. Saudou. 2022. Calcineurin and huntingtin form a calcium-sensing machinery that directs neurotrophic signals to the nucleus. Sci. Adv. 8:eabj8812. 10.1126/sciadv.abj8812 34985962 PMC8730605

[bib218] Schlager, M.A., A. Serra-Marques, I. Grigoriev, L.F. Gumy, M. Esteves da Silva, P.S. Wulf, A. Akhmanova, and C.C. Hoogenraad. 2014. Bicaudal d family adaptor proteins control the velocity of Dynein-based movements. Cell Rep. 8:1248–1256. 10.1016/j.celrep.2014.07.052 25176647

[bib219] Schmidt, L., K.E. Wain, C. Hajek, J.I. Estrada-Veras, M.J. Guillen Sacoto, I.M. Wentzensen, A. Malhotra, A. Clause, D. Perry, A. Moreno-De-Luca, and M. Bell. 2021. Expanding the phenotype of TUBB2A-related tubulinopathy: Three cases of a novel, heterozygous TUBB2A pathogenic variant p.Gly98Arg. Mol. Syndromol. 12:33–40. 10.1159/000512160 33776625 PMC7983673

[bib220] Schroeder, H.W. III, C. Mitchell, H. Shuman, E.L. Holzbaur, and Y.E. Goldman. 2010. Motor number controls cargo switching at actin-microtubule intersections in vitro. Curr. Biol. 20:687–696. 10.1016/j.cub.2010.03.024 20399098 PMC2934746

[bib221] Schumacher, J., G. Laje, R. Abou Jamra, T. Becker, T.W. Mühleisen, C. Vasilescu, M. Mattheisen, S. Herms, P. Hoffmann, A.M. Hillmer, . 2009. The DISC locus and schizophrenia: Evidence from an association study in a central European sample and from a meta-analysis across different European populations. Hum. Mol. Genet. 18:2719–2727. 10.1093/hmg/ddp204 19414483 PMC2701338

[bib222] Scott-Solomon, E., and R. Kuruvilla. 2018. Mechanisms of neurotrophin trafficking via Trk receptors. Mol. Cell. Neurosci. 91:25–33. 10.1016/j.mcn.2018.03.013 29596897 PMC6128733

[bib223] Shapira, M., R.G. Zhai, T. Dresbach, T. Bresler, V.I. Torres, E.D. Gundelfinger, N.E. Ziv, and C.C. Garner. 2003. Unitary assembly of presynaptic active zones from Piccolo-Bassoon transport vesicles. Neuron. 38:237–252. 10.1016/s0896-6273(03)00207-1 12718858

[bib224] Sharma, N., C.D. Deppmann, A.W. Harrington, C. St Hillaire, Z.Y. Chen, F.S. Lee, and D.D. Ginty. 2010. Long-distance control of synapse assembly by target-derived NGF. Neuron. 67:422–434. 10.1016/j.neuron.2010.07.018 20696380 PMC2949359

[bib225] Shin, H., M. Wyszynski, K.H. Huh, J.G. Valtschanoff, J.R. Lee, J. Ko, M. Streuli, R.J. Weinberg, M. Sheng, and E. Kim. 2003. Association of the kinesin motor KIF1A with the multimodular protein liprin-alpha. J. Biol. Chem. 278:11393–11401. 10.1074/jbc.M211874200 12522103

[bib226] Siebert, M., M.A. Böhme, J.H. Driller, H. Babikir, M.M. Mampell, U. Rey, N. Ramesh, T. Matkovic, N. Holton, S. Reddy-Alla, . 2015. A high affinity RIM-binding protein/Aplip1 interaction prevents the formation of ectopic axonal active zones. Elife. 4:e06935. 10.7554/eLife.06935 26274777 PMC4536467

[bib227] Sirajuddin, M., L.M. Rice, and R.D. Vale. 2014. Regulation of microtubule motors by tubulin isotypes and post-translational modifications. Nat. Cell Biol. 16:335–344. 10.1038/ncb2920 24633327 PMC4117587

[bib228] Sladewski, T.E., C.S. Bookwalter, M.S. Hong, and K.M. Trybus. 2013. Single-molecule reconstitution of mRNA transport by a class V myosin. Nat. Struct. Mol. Biol. 20:952–957. 10.1038/nsmb.2614 23812374 PMC3735863

[bib229] Sladewski, T.E., N. Billington, M.Y. Ali, C.S. Bookwalter, H. Lu, E.B. Krementsova, T.A. Schroer, and K.M. Trybus. 2018. Recruitment of two dyneins to an mRNA-dependent Bicaudal D transport complex. Elife. 7:e36306. 10.7554/eLife.36306 29944116 PMC6056235

[bib230] Sleigh, J.N., A.M. Rossor, A.D. Fellows, A.P. Tosolini, and G. Schiavo. 2019. Axonal transport and neurological disease. Nat. Rev. Neurol. 15:691–703. 10.1038/s41582-019-0257-2 31558780

[bib231] Song, Y., and S.T. Brady. 2015. Post-translational modifications of tubulin: Pathways to functional diversity of microtubules. Trends Cell Biol. 25:125–136. 10.1016/j.tcb.2014.10.004 25468068 PMC4344850

[bib232] Song, W., W. Li, J. Feng, L.L. Heston, W.A. Scaringe, and S.S. Sommer. 2008. Identification of high risk DISC1 structural variants with a 2% attributable risk for schizophrenia. Biochem. Biophys. Res. Commun. 367:700–706. 10.1016/j.bbrc.2007.12.117 18164685

[bib233] Splinter, D., M.E. Tanenbaum, A. Lindqvist, D. Jaarsma, A. Flotho, K.L. Yu, I. Grigoriev, D. Engelsma, E.D. Haasdijk, N. Keijzer, . 2010. Bicaudal D2, dynein, and kinesin-1 associate with nuclear pore complexes and regulate centrosome and nuclear positioning during mitotic entry. PLoS Biol. 8:e1000350. 10.1371/journal.pbio.1000350 20386726 PMC2850381

[bib234] Storbeck, M., B. Horsberg Eriksen, A. Unger, I. Hölker, I. Aukrust, L.A. Martínez-Carrera, W.A. Linke, A. Ferbert, R. Heller, M. Vorgerd, . 2017. Phenotypic extremes of BICD2-opathies: From lethal, congenital muscular atrophy with arthrogryposis to asymptomatic with subclinical features. Eur. J. Hum. Genet. 25:1040–1048. 10.1038/ejhg.2017.98 28635954 PMC5558181

[bib235] Stucchi, R., G. Plucińska, J.J.A. Hummel, E.E. Zahavi, I. Guerra San Juan, O. Klykov, R.A. Scheltema, A.F.M. Altelaar, and C.C. Hoogenraad. 2018. Regulation of KIF1A-driven dense core vesicle transport: Ca^2+^/CaM controls DCV binding and liprin-α/TANC2 recruits DCVs to postsynaptic sites. Cell Rep. 24:685–700. 10.1016/j.celrep.2018.06.071 30021165 PMC6077247

[bib236] Su, Q., Q. Cai, C. Gerwin, C.L. Smith, and Z.H. Sheng. 2004. Syntabulin is a microtubule-associated protein implicated in syntaxin transport in neurons. Nat. Cell Biol. 6:941–953. 10.1038/ncb1169 15459722

[bib237] Südhof, T.C. 2012. The presynaptic active zone. Neuron. 75:11–25. 10.1016/j.neuron.2012.06.012 22794257 PMC3743085

[bib238] Südhof, T.C. 2021. The cell biology of synapse formation. J. Cell Biol. 220:e202103052. 10.1083/jcb.202103052 34086051 PMC8186004

[bib239] Sun, T., Y. Li, T. Li, H. Ma, Y. Guo, X. Jiang, M. Hou, S. Huang, and Z. Chen. 2017. JIP1 and JIP3 cooperate to mediate TrkB anterograde axonal transport by activating kinesin-1. Cell. Mol. Life Sci. 74:4027–4044. 10.1007/s00018-017-2568-z 28638935 PMC11107601

[bib240] Suo, D., J. Park, A.W. Harrington, L.S. Zweifel, S. Mihalas, and C.D. Deppmann. 2014. Coronin-1 is a neurotrophin endosomal effector that is required for developmental competition for survival. Nat. Neurosci. 17:36–45. 10.1038/nn.3593 24270184 PMC3962792

[bib241] Sydnor, V.J., B. Larsen, D.S. Bassett, A. Alexander-Bloch, D.A. Fair, C. Liston, A.P. Mackey, M.P. Milham, A. Pines, D.R. Roalf, . 2021. Neurodevelopment of the association cortices: Patterns, mechanisms, and implications for psychopathology. Neuron. 109:2820–2846. 10.1016/j.neuron.2021.06.016 34270921 PMC8448958

[bib242] Takamori, S., M. Holt, K. Stenius, E.A. Lemke, M. Grønborg, D. Riedel, H. Urlaub, S. Schenck, B. Brügger, P. Ringler, . 2006. Molecular anatomy of a trafficking organelle. Cell. 127:831–846. 10.1016/j.cell.2006.10.030 17110340

[bib243] Takeda, S., H. Yamazaki, D.H. Seog, Y. Kanai, S. Terada, and N. Hirokawa. 2000. Kinesin superfamily protein 3 (KIF3) motor transports fodrin-associating vesicles important for neurite building. J. Cell Biol. 148:1255–1265. 10.1083/jcb.148.6.1255 10725338 PMC2174314

[bib244] Tanaka, M., J. Miyoshi, H. Ishizaki, A. Togawa, K. Ohnishi, K. Endo, K. Matsubara, A. Mizoguchi, T. Nagano, M. Sato, . 2001. Role of Rab3 GDP/GTP exchange protein in synaptic vesicle trafficking at the mouse neuromuscular junction. Mol. Biol. Cell. 12:1421–1430. 10.1091/mbc.12.5.1421 11359932 PMC34594

[bib245] Tanaka, Y., S. Niwa, M. Dong, A. Farkhondeh, L. Wang, R. Zhou, and N. Hirokawa. 2016. The molecular motor KIF1A transports the TrkA neurotrophin receptor and is essential for sensory neuron survival and function. Neuron. 90:1215–1229. 10.1016/j.neuron.2016.05.002 27263974

[bib246] Tao-Cheng, J.H. 2020. Immunogold labeling of synaptic vesicle proteins in developing hippocampal neurons. Mol. Brain. 13:9. 10.1186/s13041-020-0549-x 31959215 PMC6971973

[bib247] Taoufiq, Z., M. Ninov, A. Villar-Briones, H.Y. Wang, T. Sasaki, M.C. Roy, F. Beauchain, Y. Mori, T. Yoshida, S. Takamori, . 2020. Hidden proteome of synaptic vesicles in the mammalian brain. Proc. Natl. Acad. Sci. USA. 117:33586–33596. 10.1073/pnas.2011870117 33376223 PMC7776996

[bib248] Tas, R.P., A. Chazeau, B.M.C. Cloin, M.L.A. Lambers, C.C. Hoogenraad, and L.C. Kapitein. 2017. Differentiation between oppositely oriented microtubules controls polarized neuronal transport. Neuron. 96:1264–1271.e5. 10.1016/j.neuron.2017.11.018 29198755 PMC5746200

[bib249] Teng, J., T. Rai, Y. Tanaka, Y. Takei, T. Nakata, M. Hirasawa, A.B. Kulkarni, and N. Hirokawa. 2005. The KIF3 motor transports N-cadherin and organizes the developing neuroepithelium. Nat. Cell Biol. 7:474–482. 10.1038/ncb1249 15834408

[bib250] Terenzio, M., M. Golding, M.R.G. Russell, K.B. Wicher, I. Rosewell, B. Spencer-Dene, D. Ish-Horowicz, and G. Schiavo. 2014. Bicaudal-D1 regulates the intracellular sorting and signalling of neurotrophin receptors. EMBO J. 33:1582–1598. 10.15252/embj.201387579 24920579 PMC4198053

[bib251] Terenzio, M., G. Schiavo, and M. Fainzilber. 2017. Compartmentalized signaling in neurons: From cell biology to neuroscience. Neuron. 96:667–679. 10.1016/j.neuron.2017.10.015 29096079

[bib252] Thapar, A., M. Cooper, and M. Rutter. 2017. Neurodevelopmental disorders. Lancet Psychiatry. 4:339–346. 10.1016/S2215-0366(16)30376-5 27979720

[bib253] Toba, S., Y. Tamura, K. Kumamoto, M. Yamada, K. Takao, S. Hattori, T. Miyakawa, Y. Kataoka, M. Azuma, K. Hayasaka, . 2013. Post-natal treatment by a blood-brain-barrier permeable calpain inhibitor, SNJ1945 rescued defective function in lissencephaly. Sci. Rep. 3:1224. 10.1038/srep01224 23390575 PMC3565454

[bib254] Toda, H., H. Mochizuki, R. Flores III, R. Josowitz, T.B. Krasieva, V.J. Lamorte, E. Suzuki, J.G. Gindhart, K. Furukubo-Tokunaga, and T. Tomoda. 2008. UNC-51/ATG1 kinase regulates axonal transport by mediating motor-cargo assembly. Genes Dev. 22:3292–3307. 10.1101/gad.1734608 19056884 PMC2600757

[bib255] Tomishige, M., D.R. Klopfenstein, and R.D. Vale. 2002. Conversion of Unc104/KIF1A kinesin into a processive motor after dimerization. Science. 297:2263–2267. 10.1126/science.1073386 12351789

[bib256] Trimouille, A., É. Obre, G. Banneau, A. Durr, G. Stevanin, F. Clot, P. Pennamen, J.T. Perez, C. Bailly-Scappaticci, M. Rouanet, . 2018. An inframe deletion in BICD2 associated with a non-progressive form of SMALED. Clin. Neurol. Neurosurg. 166:1–3. 10.1016/j.clineuro.2018.01.013 29353221

[bib257] Truckenbrodt, S., A. Viplav, S. Jähne, A. Vogts, A. Denker, H. Wildhagen, E.F. Fornasiero, and S.O. Rizzoli. 2018. Newly produced synaptic vesicle proteins are preferentially used in synaptic transmission. EMBO J. 37:e98044. 10.15252/embj.201798044 29950309 PMC6068464

[bib258] Tsurusaki, Y., S. Saitoh, K. Tomizawa, A. Sudo, N. Asahina, H. Shiraishi, J. Ito, H. Tanaka, H. Doi, H. Saitsu, . 2012. A DYNC1H1 mutation causes a dominant spinal muscular atrophy with lower extremity predominance. Neurogenetics. 13:327–332. 10.1007/s10048-012-0337-6 22847149

[bib259] Turner, T.J., C. Zourray, S. Schorge, and G. Lignani. 2021. Recent advances in gene therapy for neurodevelopmental disorders with epilepsy. J. Neurochem. 157:229–262. 10.1111/jnc.15168 32880951 PMC8436749

[bib260] Turner-Bridger, B., M. Jakobs, L. Muresan, H.H. Wong, K. Franze, W.A. Harris, and C.E. Holt. 2018. Single-molecule analysis of endogenous β-actin mRNA trafficking reveals a mechanism for compartmentalized mRNA localization in axons. Proc. Natl. Acad. Sci. USA. 115:E9697–E9706. 10.1073/pnas.1806189115 30254174 PMC6187124

[bib261] Tushev, G., C. Glock, M. Heumüller, A. Biever, M. Jovanovic, and E.M. Schuman. 2018. Alternative 3′ UTRs modify the localization, regulatory potential, stability, and plasticity of mRNAs in neuronal compartments. Neuron. 98:495–511.e6. 10.1016/j.neuron.2018.03.030 29656876

[bib262] Valence, S., K. Poirier, N. Lebrun, Y. Saillour, P. Sonigo, B. Bessières, T. Attié-Bitach, A. Benachi, C. Masson, F. Encha-Razavi, . 2013. Homozygous truncating mutation of the KBP gene, encoding a KIF1B-binding protein, in a familial case of fetal polymicrogyria. Neurogenetics. 14:215–224. 10.1007/s10048-013-0373-x 24072599

[bib263] van den Pol, A.N. 2012. Neuropeptide transmission in brain circuits. Neuron. 76:98–115. 10.1016/j.neuron.2012.09.014 23040809 PMC3918222

[bib264] Vasudevan, A., N. Ratnakaran, K. Murthy, S. Kumari, D.H. Hall, and S.P. Koushika. 2024. Preferential transport of synaptic vesicles across neuronal branches is regulated by the levels of the anterograde motor UNC-104/KIF1A in vivo. Genetics:iyae021. 10.1093/genetics/iyae021 38467475 PMC11232277

[bib265] Verhey, K.J., D. Meyer, R. Deehan, J. Blenis, B.J. Schnapp, T.A. Rapoport, and B. Margolis. 2001. Cargo of kinesin identified as JIP scaffolding proteins and associated signaling molecules. J. Cell Biol. 152:959–970. 10.1083/jcb.152.5.959 11238452 PMC2198804

[bib266] Vissers, L.E., J. de Ligt, C. Gilissen, I. Janssen, M. Steehouwer, P. de Vries, B. van Lier, P. Arts, N. Wieskamp, M. del Rosario, . 2010. A de novo paradigm for mental retardation. Nat. Genet. 42:1109–1112. 10.1038/ng.712 21076407

[bib267] Vitet, H., J. Bruyère, H. Xu, C. Séris, J. Brocard, Y.S. Abada, B. Delatour, C. Scaramuzzino, L. Venance, and F. Saudou. 2023. Huntingtin recruits KIF1A to transport synaptic vesicle precursors along the mouse axon to support synaptic transmission and motor skill learning. Elife. 12:e81011. 10.7554/eLife.81011 37431882 PMC10365837

[bib268] Vukoja, A., U. Rey, A.G. Petzoldt, C. Ott, D. Vollweiter, C. Quentin, D. Puchkov, E. Reynolds, M. Lehmann, S. Hohensee, . 2018. Presynaptic biogenesis requires axonal transport of lysosome-related vesicles. Neuron. 99:1216–1232.e7. 10.1016/j.neuron.2018.08.004 30174114

[bib269] Weedon, M.N., R. Hastings, R. Caswell, W. Xie, K. Paszkiewicz, T. Antoniadi, M. Williams, C. King, L. Greenhalgh, R. Newbury-Ecob, and S. Ellard. 2011. Exome sequencing identifies a DYNC1H1 mutation in a large pedigree with dominant axonal Charcot-Marie-Tooth disease. Am. J. Hum. Genet. 89:308–312. 10.1016/j.ajhg.2011.07.002 21820100 PMC3155164

[bib270] Willemsen, M.H., L.E. Vissers, M.A. Willemsen, B.W. van Bon, T. Kroes, J. de Ligt, B.B. de Vries, J. Schoots, D. Lugtenberg, B.C. Hamel, . 2012. Mutations in DYNC1H1 cause severe intellectual disability with neuronal migration defects. J. Med. Genet. 49:179–183. 10.1136/jmedgenet-2011-100542 22368300

[bib271] Wong, M.Y., C. Zhou, D. Shakiryanova, T.E. Lloyd, D.L. Deitcher, and E.S. Levitan. 2012. Neuropeptide delivery to synapses by long-range vesicle circulation and sporadic capture. Cell. 148:1029–1038. 10.1016/j.cell.2011.12.036 22385966 PMC3294265

[bib272] Wu, Y.E., L. Huo, C.I. Maeder, W. Feng, and K. Shen. 2013. The balance between capture and dissociation of presynaptic proteins controls the spatial distribution of synapses. Neuron. 78:994–1011. 10.1016/j.neuron.2013.04.035 23727120 PMC3898717

[bib273] Wu, X., Q. Cai, Z. Shen, X. Chen, M. Zeng, S. Du, and M. Zhang. 2019. RIM and RIM-BP form presynaptic active-zone-like condensates via phase separation. Mol. Cell. 73:971–984.e5. 10.1016/j.molcel.2018.12.007 30661983

[bib274] Xie, Y., B. Zhou, M.Y. Lin, S. Wang, K.D. Foust, and Z.H. Sheng. 2015. Endolysosomal deficits augment mitochondria pathology in spinal motor neurons of asymptomatic fALS mice. Neuron. 87:355–370. 10.1016/j.neuron.2015.06.026 26182418 PMC4511489

[bib275] Xiong, G.J., X.T. Cheng, T. Sun, Y. Xie, N. Huang, S. Li, M.Y. Lin, and Z.H. Sheng. 2021. Defects in syntabulin-mediated synaptic cargo transport associate with autism-like synaptic dysfunction and social behavioral traits. Mol. Psychiatry. 26:1472–1490. 10.1038/s41380-020-0713-9 32332993 PMC7584772

[bib276] Xu, F., H. Takahashi, Y. Tanaka, S. Ichinose, S. Niwa, M.P. Wicklund, and N. Hirokawa. 2018. KIF1Bβ mutations detected in hereditary neuropathy impair IGF1R transport and axon growth. J. Cell Biol. 217:3480–3496. 10.1083/jcb.201801085 30126838 PMC6168269

[bib277] Yamada, M., Y. Yoshida, D. Mori, T. Takitoh, M. Kengaku, H. Umeshima, K. Takao, T. Miyakawa, M. Sato, H. Sorimachi, . 2009. Inhibition of calpain increases LIS1 expression and partially rescues in vivo phenotypes in a mouse model of lissencephaly. Nat. Med. 15:1202–1207. 10.1038/nm.2023 19734909 PMC2759411

[bib278] Yamashita, N. 2019. Retrograde signaling via axonal transport through signaling endosomes. J. Pharmacol. Sci. 141:91–96. 10.1016/j.jphs.2019.10.001 31679963

[bib279] Yonekawa, Y., A. Harada, Y. Okada, T. Funakoshi, Y. Kanai, Y. Takei, S. Terada, T. Noda, and N. Hirokawa. 1998. Defect in synaptic vesicle precursor transport and neuronal cell death in KIF1A motor protein-deficient mice. J. Cell Biol. 141:431–441. 10.1083/jcb.141.2.431 9548721 PMC2148442

[bib280] Yoshihara, S., X. Jiang, M. Morikawa, T. Ogawa, S. Ichinose, H. Yabe, A. Kakita, M. Toyoshima, Y. Kunii, T. Yoshikawa, . 2021. Betaine ameliorates schizophrenic traits by functionally compensating for KIF3-based CRMP2 transport. Cell Rep. 35:108971. 10.1016/j.celrep.2021.108971 33852848

[bib281] Youn, Y.H., T. Pramparo, S. Hirotsune, and A. Wynshaw-Boris. 2009. Distinct dose-dependent cortical neuronal migration and neurite extension defects in Lis1 and Ndel1 mutant mice. J. Neurosci. 29:15520–15530. 10.1523/JNEUROSCI.4630-09.2009 20007476 PMC2824645

[bib282] Zablotsky, B., L.I. Black, M.J. Maenner, L.A. Schieve, M.L. Danielson, R.H. Bitsko, S.J. Blumberg, M.D. Kogan, and C.A. Boyle. 2019. Prevalence and trends of developmental disabilities among children in the United States: 2009–2017. Pediatrics. 144:e20190811. 10.1542/peds.2019-0811 31558576 PMC7076808

[bib283] Zahavi, E.E., J.J.A. Hummel, Y. Han, C. Bar, R. Stucchi, M. Altelaar, and C.C. Hoogenraad. 2021. Combined kinesin-1 and kinesin-3 activity drives axonal trafficking of TrkB receptors in Rab6 carriers. Dev. Cell. 56:494–508.e7. 10.1016/j.devcel.2021.01.010 33571451 PMC7907685

[bib284] Zhai, R., G. Olias, W.J. Chung, R.A.J. Lester, S. tom Dieck, K. Langnaese, M.R. Kreutz, S. Kindler, E.D. Gundelfinger, and C.C. Garner. 2000. Temporal appearance of the presynaptic cytomatrix protein bassoon during synaptogenesis. Mol. Cell. Neurosci. 15:417–428. 10.1006/mcne.2000.0839 10833299

[bib285] Zhai, R.G., H. Vardinon-Friedman, C. Cases-Langhoff, B. Becker, E.D. Gundelfinger, N.E. Ziv, and C.C. Garner. 2001. Assembling the presynaptic active zone: A characterization of an active one precursor vesicle. Neuron. 29:131–143. 10.1016/s0896-6273(01)00185-4 11182086

[bib286] Zhang, B., J. Carroll, J.Q. Trojanowski, Y. Yao, M. Iba, J.S. Potuzak, A.M. Hogan, S.X. Xie, C. Ballatore, A.B. Smith III, . 2012. The microtubule-stabilizing agent, epothilone D, reduces axonal dysfunction, neurotoxicity, cognitive deficits, and Alzheimer-like pathology in an interventional study with aged tau transgenic mice. J. Neurosci. 32:3601–3611. 10.1523/JNEUROSCI.4922-11.2012 22423084 PMC3321513

[bib287] Zhao, C., J. Takita, Y. Tanaka, M. Setou, T. Nakagawa, S. Takeda, H.W. Yang, S. Terada, T. Nakata, Y. Takei, . 2001. Charcot-Marie-Tooth disease type 2A caused by mutation in a microtubule motor KIF1Bbeta. Cell. 105:587–597. 10.1016/s0092-8674(01)00363-4 11389829

[bib288] Zhou, B., Y.B. Zhu, L. Lin, Q. Cai, and Z.H. Sheng. 2011. Snapin deficiency is associated with developmental defects of the central nervous system. Biosci. Rep. 31:151–158. 10.1042/BSR20100110 20946101 PMC4957243

[bib289] Zhou, B., Q. Cai, Y. Xie, and Z.H. Sheng. 2012. Snapin recruits dynein to BDNF-TrkB signaling endosomes for retrograde axonal transport and is essential for dendrite growth of cortical neurons. Cell Rep. 2:42–51. 10.1016/j.celrep.2012.06.010 22840395 PMC3408618

